# Monocyte state 1 (MS1) cells in critically ill patients with sepsis or non-infectious conditions: association with disease course and host response

**DOI:** 10.1186/s13054-024-04868-5

**Published:** 2024-03-19

**Authors:** Giuseppe G. F. Leite, Justin de Brabander, Erik H. A. Michels, Joe M. Butler, Olaf L. Cremer, Brendon P. Scicluna, Timothy E. Sweeney, Miguel Reyes, Reinaldo Salomao, Hessel Peters-Sengers, Tom van der Poll

**Affiliations:** 1 Center for Experimental and Molecular Medicine (CEMM), Amsterdam UMC, Location University of Amsterdam, Meibergdreef 9, 1105 AZ Amsterdam, The Netherlands; 2 Division of Infectious Diseases, Department of Medicine, Escola Paulista de Medicina, Universidade Federal de São Paulo, São Paulo, Brazil; 3 Department of Intensive Care Medicine, UMC Utrecht, Utrecht University, Utrecht, The Netherlands; 4 Department of Applied Biomedical Science, Faculty of Health Sciences, Mater Dei Hospital, University of Malta, Msida, Malta; 5 Centre for Molecular Medicine and Biobanking, University of Malta, Msida, Malta; 6 Inflammatix Inc., Sunnyvale, CA USA; 7 Department of Infectious Diseases, Genentech, South San Francisco, USA; 8 Department of Epidemiology and Data Science, Amsterdam UMC, Location Vrije Universiteit, Amsterdam, The Netherlands; 9 Division of Infectious Diseases, Amsterdam UMC, Location University of Amsterdam, Amsterdam, The Netherlands

**Keywords:** Myeloid-derived suppressor cells, Shock, Hyperinflammation and immune suppression

## Abstract

**Background:**

Sepsis is a life-threatening condition arising from an aberrant host response to infection. Recent single-cell RNA sequencing investigations identified an immature bone-marrow-derived CD14^+^ monocyte phenotype with immune suppressive properties termed “monocyte state 1” (MS1) in patients with sepsis. Our objective was to determine the association of MS1 cell profiles with disease presentation, outcomes, and host response characteristics.

**Methods:**

We used the transcriptome deconvolution method (CIBERSORTx) to estimate the percentage of MS1 cells from blood RNA profiles of patients with sepsis admitted to the intensive care unit (ICU). We compared these profiles to ICU patients without infection and to healthy controls. Host response dysregulation was further studied by gene co-expression network and gene set enrichment analyses of blood leukocytes, and measurement of 15 plasma biomarkers indicative of pathways implicated in sepsis pathogenesis.

**Results:**

Sepsis patients (*n* = 332) were divided into three equally-sized groups based on their MS1 cell levels (low, intermediate, and high). MS1 groups did not differ in demographics or comorbidities. The intermediate and high MS1 groups presented with higher disease severity and more often had shock. MS1 cell abundance did not differ between survivors and non-survivors, or between patients who did or did not acquire a secondary infection. Higher MS1 cell percentages were associated with downregulation of lymphocyte-related and interferon response genes in blood leukocytes, with concurrent upregulation of inflammatory response pathways, including tumor necrosis factor signaling via nuclear factor-κB. Previously described sepsis host response transcriptomic subtypes showed different MS1 cell abundances, and MS1 cell percentages positively correlated with the “quantitative sepsis response signature” and “molecular degree of perturbation” scores. Plasma biomarker levels, indicative of inflammation, endothelial cell activation, and coagulation activation, were largely similar between MS1 groups. In ICU patients without infection (*n* = 215), MS1 cell percentages and their relation with disease severity, shock, and host response dysregulation were highly similar to those in sepsis patients.

**Conclusions:**

High MS1 cell percentages are associated with increased disease severity and shock in critically ill patients with sepsis or a non-infectious condition. High MS1 cell abundance likely indicates broad immune dysregulation, entailing not only immunosuppression but also anomalies reflecting exaggerated inflammatory responses.

**Graphical abstract:**

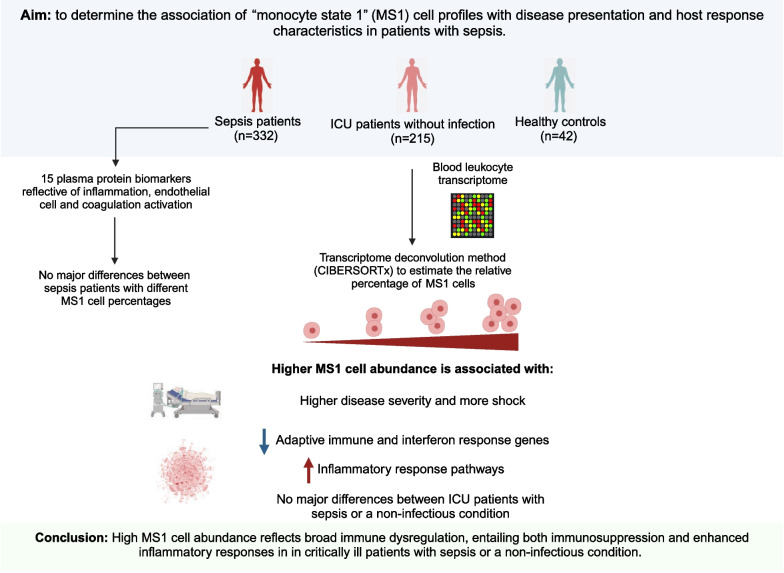

**Supplementary Information:**

The online version contains supplementary material available at 10.1186/s13054-024-04868-5.

## Introduction

Sepsis is a life-threatening condition resulting from a dysregulated host response to infection [1]. Sepsis and septic shock continue to represent significant risks for mortality in critically ill patients [2]. In 2017, there were approximately 49 million new cases of sepsis and 11 million sepsis-related deaths worldwide [3].

The pathophysiology of sepsis is complex, encompassing a variety of proinflammatory and immunosuppressive responses [4]. Myeloid-derived suppressor cells (MDSCs) have been implicated in sepsis-induced immune suppression [5]. Two main subpopulations are usually considered: polymorphonuclear MDSCs (PMN-MDSCs) and monocytic MDSCs (M-MDSCs) [5]. MDSCs have the capacity to hinder immune responses, encompassing those modulated by T cells, B cells, and natural killer (NK) cells. PMN-MDSCs and M-MDSCs share critical biochemical attributes that enable the suppression of immune responses [6].

Recent studies applied single-cell RNA sequencing (scRNA-seq) to understand the spectrum of immune cell states in the blood of sepsis patients [7–9]. scRNA-seq has identified an immature bone-marrow-derived CD14^+^ monocyte phenotype, denoted as “monocyte state 1” (MS1), which is reminiscent of M-MDSCs [7, 8]. This monocyte phenotype is characterized by elevated expression levels of *RETN*, *ALOX5AP* and *IL1R2*, and reduced expression of class II major histocompatibility complex (MHC-II). MS1 cells can be induced from bone marrow precursors, and display several immunosuppressive properties, including suppression of T cell proliferation and inhibition of the inflammatory activation of epithelial and endothelial cells [7, 8]. Furthermore, an independent investigation reported the presence of a neutrophil subset, designated as “IL1R2^+^ Neu” in sepsis patients [9]. These cells exhibit gene expression profiles remarkably similar to those of MS1 cells, suggesting that common myelopoietic processes might underlie the development of both MS1 cells and IL1R2^+^ Neu cells [9].

The proportion of MS1 cells can be estimated by deconvolution of bulk RNA expression data from whole blood [7, 8, 10]. In this study, we leveraged this validated deconvolution method to evaluate the percentage of MS1 cells using whole blood transcriptome data from a well-characterized cohort of sepsis patients. By doing so, we aimed to determine the association of MS1 cell profiles with disease presentation, outcomes and host response characteristics, using non-infected critically ill patients and healthy individuals as comparators.

## Methods

### Study design and population

This study was conducted as part of the Molecular Diagnosis and Risk Stratification of Sepsis (MARS) project (ClinicalTrials.gov identifier NCT01905033), a prospective observational study conducted in two tertiary hospitals in the Netherlands between January 2011 and January 2014 (Academic Medical Center, Amsterdam, and University Medical Center Utrecht, Utrecht) [11]. For this investigation, we enrolled consecutive patients who received a sepsis diagnosis within 24 h of admission to the intensive care unit (ICU). The sepsis definition used was based on the Sepsis-3 criteria [1]; patients were retrospectively classified as meeting these criteria using prospectively collected data. In additional analyses, we compared the percentage of MS1 cells of patients with sepsis to those admitted to the ICU for non-infectious conditions. We excluded patients who were readmitted or transferred from another ICU, unless the transfer occurred on the initial day of ICU presentation. For definitions of comorbidities, organ dysfunctions and complications, see Additional file 1. Furthermore, we analyzed transcriptomic data from healthy controls (Gene Expression Omnibus access number: GSE65682).

### Measurements

For detailed information on microarray experiments, RNA processing protocols, plasma biomarker assays, and the corresponding analysis methods, please refer to the methods provided in Additional file 1.

### Bulk data deconvolution

We utilized CIBERSORTx [10] to estimate the percentage of MS1 cells from the bulk normalized gene expression matrix. To deconvolute the whole blood gene expression data, we used the cell state signature matrix generated from scRNA-seq of peripheral blood mononuclear cells as reference [7]. The matrix was previously optimized to finding the minimum number of genes where the reduction in prediction error is saturated. This signature matrix encompasses 16 immune cell states. CIBERSORTx was performed with batch correction, quantile normalization, absolute mode, and 100 permutations. Patients were ranked by the percentage of MS1 cells and then divided into tertiles—three groups of similar size—corresponding to low, intermediate and high percentage of MS1 cells. As a sensitivity analysis, we also stratified patients applying one-dimensional k-means clustering based on MS1 cell percentages [12]; the optimal number of clusters was determined using a consensus-based algorithm.

### Bioinformatics

We conducted gene co-expression network and module analysis, correlation with gene expression matrix, and correlation with other molecular signatures (see Additional file 1 for details).

### Statistical analysis

Statistical analyses were performed using R (version 4.3.0). Normality was assessed using the Shapiro–Wilk test and Quantile–Quantile plots, and all data showed non-normal distribution. Variables are reported as median values with 25th and 75th percentiles. Comparisons between groups were performed using either the Mann–Whitney *U* test or the Kruskal–Wallis test, followed by Dunn's test with Benjamini–Hochberg (BH) correction for multiple comparisons. For categorical data, the chi-squared test was utilized for comparison. Host response plasma biomarkers were stratified into three pathophysiological domains (inflammatory response, endothelial cell activation, and coagulation activation) as described [13–15]. To visualize the overall differences among the plasma biomarkers between MS1 groups a principal component analysis (PCA) was conducted, following previously established methods [13, 14]. Briefly, prior to PCA, the plasma biomarker data were centered and scaled to unit variance. Subsequently, differences in the principal component (PC) scores between MS1 groups were analyzed using analysis of variance. Differences in individual biomarker levels between the three MS1 groups were quantified using the Hedges’ g effect size and visualized using heatmaps [16]. The very few missing biomarkers were imputed by random forest, with the function rfImpute in the randomForest package in R. Differences in 30-day survival were visualized by Kaplan–Meier survival curves. Additionally, in a regression analysis exploring the risk of 30-day mortality with MS1 cell percentage modeled as a continuous variable, we employed a restricted cubic spline function with three inner knots at default quantile locations. We also estimated the risk of ICU-acquired infections with the cumulative incidence function, which takes account of ICU death and ICU discharge as competing risks, comparing cumulative incidence curves among the three MS1 groups (Gray's competing-risks analysis).

## Results

### Association of MS1 percentage with clinical presentation and outcome in patients with sepsis

We determined the percentage of MS1 cells in 332 patients diagnosed with sepsis and stratified these into tertiles based on their relative abundance of MS1 cells: low, intermediate, and high (Additional file 1: Fig. S1; Table 1). The range of the MS1 cell proportions aligned with findings in prior studies [7, 8]. MS1 groups were largely similar regarding demographics, chronic comorbidities, and site of infection, although the proportion of abdominal infections was higher in the intermediate and high MS1 groups, as compared with the low MS1 group. These groups also presented with a higher disease severity upon ICU admission, as indicated by higher sequential organ failure assessment (SOFA) scores, Acute Physiology and Chronic Health Evaluation (APACHE) IV scores, and Acute Physiology Scores (APS), as well as higher frequencies of acute kidney injury (AKI) and shock. Length of ICU or hospital stay did not differ between MS1 groups.

**Table 1:** Baseline characteristics and outcomes of patients admitted to the ICU with sepsis stratified into tertiles by percentage of MS1 cells

Characteristic	Low (*N* = 111)^a^	Intermediate (*N* = 111)^a^	High (*N* = 110)^a^	*p* value
Percentage of MS1 cells	19.0 (17.4, 20.2)^b^	23.7 (22.6, 25.0)^c^	30.3 (27.9, 34.0)^d^	**< 0.00001**
Demographics				
Age years	62.0 (49.0, 72.5)	65.0 (56.0, 74.0)	64.5 (56.0, 75.0)	0.2
White race	88 (79%)	96 (86%)	98 (89%)	0.1
Male sex	70 (63%)	62 (56%)	65 (59%)	0.5
Admission type, surgery	25 (23%)	39 (35%)	27 (25%)	0.07
BMI	24.8 (22.5, 29.4)^b,c^	25.7 (23.1, 29.5)^b^	24.0 (21.7, 27.3)^c^	**0.04**
Comorbidity				
Charlson score	3.0 (2.0, 5.0)	4.0 (3.0, 6.0)	4.0 (2.0, 5.0)	0.2
Cardiovascular insufficiency	3 (2.7%)	7 (6.3%)	6 (5.5%)	0.4
Respiratory insufficiency	9 (8.1%)	6 (5.4%)	9 (8.2%)	0.7
Renal insufficiency	13 (12%)	14 (13%)	11 (10%)	0.8
Hypertension	35 (32%)	32 (29%)	22 (20%)	0.1
Diabetes mellitus	21 (19%)	20 (18%)	18 (16%)	0.9
COPD	16 (14%)	16 (14%)	17 (15%)	1.0
Cerebrovascular disease	6 (5.4%)	13 (12%)	8 (7.3%)	0.2
Site of infection				
Respiratory	47 (42%)	35 (32%)	40 (36%)	0.2
Abdominal	14 (13%)^b^	24 (22%)^b,c^	32 (29%)^c^	**0.01**
Cardiovascular	8 (7.2%)	6 (5.4%)	2 (1.8%)	0.2
Urinary	6 (5.4%)	7 (6.3%)	11 (10%)	0.4
CNS	0 (0%)	3 (2.7%)	1 (0.9%)	0.2
Skin	6 (5.4%)	5 (4.5%)	6 (5.5%)	> 0.9
Other	5 (4.5%)	5 (4.5%)	0 (0%)	0.1
Unknown	1 (0.9%)	2 (1.8%)	2 (1.8%)	0.8
Mix infection	24 (22%)	24 (22%)	16 (15%)	0.3
Disease severity on admission				
SOFA Score	6.0 (4.0, 9.0)^b^	8.0 (5.0, 10.0)^c^	7.5 (5.0, 10.0)^c^	**0.005**
APACHE IV score	73.0 (55.0, 88.5)^b^	84.0 (66.0, 104.5)^c^	81.0 (63.3, 103.8)^c^	**0.003**
APS	60.0 (48.0, 77.0)^b^	71.0 (56.0, 91.0)^c^	69.5 (50.0, 90.8)^c^	**0.01**
ARDS	23 (21%)	36 (32%)	31 (28%)	0.1
AKI	32 (29%)^b^	52 (47%)^c^	43 (39%)^b,c^	**0.02**
Septic shock	29 (26%)^b^	44 (40%)^b,c^	51 (46%)^c^	**0.01**
Outcomes				
Hospital LOS, days	14.0 (8.0, 35.0)	17.0 (8.5, 36.5)	15.5 (7.3, 39.5)	0.9
ICU LOS, days	4.0 (2.0, 10.0)	5.0 (3.0, 11.0)	6.0 (2.0, 11.0)	0.2
ICU-acquired complications				
ARDS	6 (5.4%)^b^	17 (15%)^c^	18 (16%)^c^	**0.02**
Infection	10 (9.0%)	11 (9.9%)	18 (16%)	0.2
Mortality				
Death in ICU	19 (17%)	24 (22%)	23 (21%)	0.7
30‐day mortality	26 (23%)	34 (31%)	33 (30%)	0.4
60‐day mortality	31 (28%)	38 (34%)	40 (36%)	0.3
90‐day mortality	32 (29%)	40 (36%)	45 (41%)	0.2

Bold indicates that the values are statistically significant

*AKI* acute kidney injury, *APACHE* acute physiology and chronic health evaluation, *APS* acute physiology scores, *ARDS* acute respiratory distress syndrome, *BMI* body mass index, *COPD* chronic obstructive pulmonary disease, *CNS* central nervous system, *LOS* length of stay, *SOFA* sequential organ failure assessment

^a^Median (IQR); *n*/*N* (%)

^b–d^Groups that have no superscript in common are significantly different from each other after post-hoc tests with Benjamini–Hochberg correction (*p* < 0.05)

Interestingly, patients with abdominal infections in our cohort presented higher disease severity upon ICU admission, as indicated by higher SOFA scores, and increased frequencies of AKI and shock compared to patients with respiratory infections (Additional file 1: Table S1). In a sensitivity analysis, adjusting for disease severity on admission (SOFA, APACHE IV APS score, shock, ARDS, and AKI) in a logistic regression model, the proportion of MS1 cells did not differ anymore between patients with abdominal and respiratory infections (adjusted *p* value = 0.31). Additionally, we directly compared patients with abdominal infections and shock to those with respiratory infections and shock. This comparison showed no statistically significant differences in MS1 cell percentages or severity scores (Additional file 1: Table S2), suggesting that the severity of the disease, rather than the infection source, predominantly impacts MS1 cell proportions.

No overall differences were observed in the proportion of surviving patients between the three MS1 groups (log-rank, *p* = 0.43) (Additional file 1: Fig. S2A). Mortality rates showed a nonlinear relationship with MS1 cell percentages (Additional file 1: Fig. S2B); according to this model the increase in MS1 levels did not have a significant impact on the probability of the 30-day mortality event (*p* = 0.13).

The intermediate and high MS1 groups more frequently developed ARDS while in the ICU. The frequency of secondary ICU-acquired infections was comparable between MS1 groups. We also assessed the risk of ICU-acquired infections with the cumulative incidence function. Patients classified in the high MS1 group exhibited the highest cumulative incidence of ICU-acquired infections; however, no overall differences between MS1 groups were observed (Gray's Test, *p* = 0.2) (Additional file 1: Fig. S3).

To test the robustness of our results, we attempted to stratify the cohort using a different methodology. This involved conducting a sensitivity analysis using one-dimensional k-means clustering based on MS1 cell percentages. The optimal number of clusters was determined to be 2 clusters, supported by 11 out of 28 methods (Additional file 1: Table S3). This analysis yielded results similar to those observed in the comparison of MS1 cell tertiles, with the group of patients classified as cluster 2 (high MS1 cell abundance) showing a higher incidence of sepsis due to abdominal infections, higher disease severity upon ICU admission, and a more frequent development of ARDS while in the ICU (Additional file 1: Table S4).

### Weighted gene co-expression network analysis

To obtain a first insight into differences in the blood transcriptomes across patients with different MS1 cell abundances, we determined the number of differentially expressed genes between the three MS1 groups in pair-wise comparisons (Additional file 1: Fig. S4). The low MS1 group was clearly distinct from the intermediate and high MS1 groups.

**Fig. 1:**
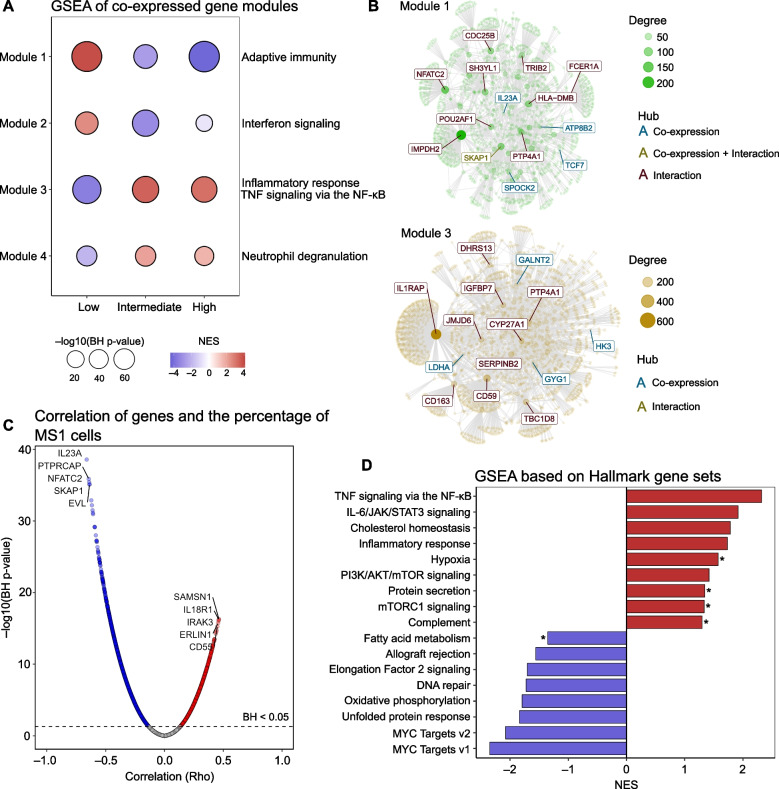
Co-expressed gene modules and gene set enrichment analysis in patients with sepsis stratified according to MS1 cell percentages in blood. **A** The co-expression module identification analysis revealed distinct gene modules based on MS1 cell levels in patients with sepsis. Patients were categorized into low MS1 (*n* = 111), intermediate MS1 (*n* = 111), and high MS1 groups (*n* = 110). The size of each circle in the graph is proportional to − log10(BH adjusted *p* value), and the color represents the normalized enrichment score (NES). Blue indicates a decreased NES, and red color represents an increased NES. **B** The network diagrams depict the two largest differential co-expression gene modules (Module 2 and Module 3) identified in the analysis; the network highlights the hub genes, which are crucial regulatory genes within each module, **C** The graph illustrates the correlation between the percentage of MS1 cells and the normalized gene expression matrix. Positive correlations with a BH adjusted *p* value < 0.05 are shown in red, negative correlations in blue; and non-significant correlations are depicted in gray. Gene names indicate the top five positively correlated (in red) and the top five negatively correlated (in blue) genes. **D** Gene set enrichment analysis performed on genes that correlated with MS1 levels. The color-coded NES values signify the enrichment score for each pathway; all pathways displayed a BH adjusted *p* value < 0.05, except for pathways marked by an asterisk (*), which indicates a BH adjusted *p* value < 0.1

To investigate subgroup-specific transcriptional regulation in sepsis patients based on the percentage of MS1 cells, we conducted a comprehensive gene co-expression network analysis followed by an unbiased overrepresentation analysis. This analysis revealed nine gene expression modules, of which four modules (Fig. 1A) were significantly different between the three groups (by Kruskal–Wallis test comparing the modules’ eigengene) and overrepresented with distinct biological pathways (Additional file 1: Table S5). Modules 1 and 2 exhibited increased normalized enrichment scores (NES) in the low MS1 group. Module 1 demonstrated significant enrichment in pathways related to the adaptive immune system, while Module 2 displayed enrichments primarily linked to interferon signaling. On the other hand, Modules 3 and 4, which showed elevated NES in the intermediate and high MS1 groups, were associated with inflammatory response and tumor necrosis factor (TNF) signaling via nuclear factor-κB, as well as with neutrophil degranulation, respectively (Fig. 1A). To identify potential key regulatory genes governing these pathways, we focused on the central hub genes within each module (most connected genes). In the network of Module 1, several lymphocyte-related genes emerged as hubs, including *IL23A, TCF7, SKAP1, IMPDH2,* and *NFATC2* (Fig. 1B). In the network of Module 3, hub genes of interest comprised *CD163*, *CD59*, *IL1RAP*, *JMJD6*, *LDHA*, *HK3*, and *GYG1*.

Next, we utilized the proportion of MS1 cells on a continuous scale and performed a functional enrichment analysis based on the correlation value (rho) with the genes in the normalized expression matrix. These results corroborated the findings observed in the module analysis (Fig. 1C). Moreover, this analysis uncovered additional enriched pathways that were not evident in the module analysis. Specifically, pathways with increased NES included IL-6/JAK/STAT3 signaling, hypoxia, mTORC1 signaling, and complement. Pathways with negative NES consisted of Elongation Factor (EF)-2 signaling, oxidative phosphorylation, and unfolded protein response (Fig. 1D).

Comparison of the two MS1 clusters derived by k-means clustering (Additional file 1: Table S4) resulted in similar differential gene expression patterns between MS1 groups. For instance, cluster 2 (high MS1 cell abundance) exhibited an elevated expression of genes associated with inflammatory and innate immune responses, along with a decreased expression of genes related to adaptive immunity (Additional file 1: Fig. S5 and Table S6).

### Distinctive and overlapping host response biomarker profiles in MS1 groups

**Fig. 2:**
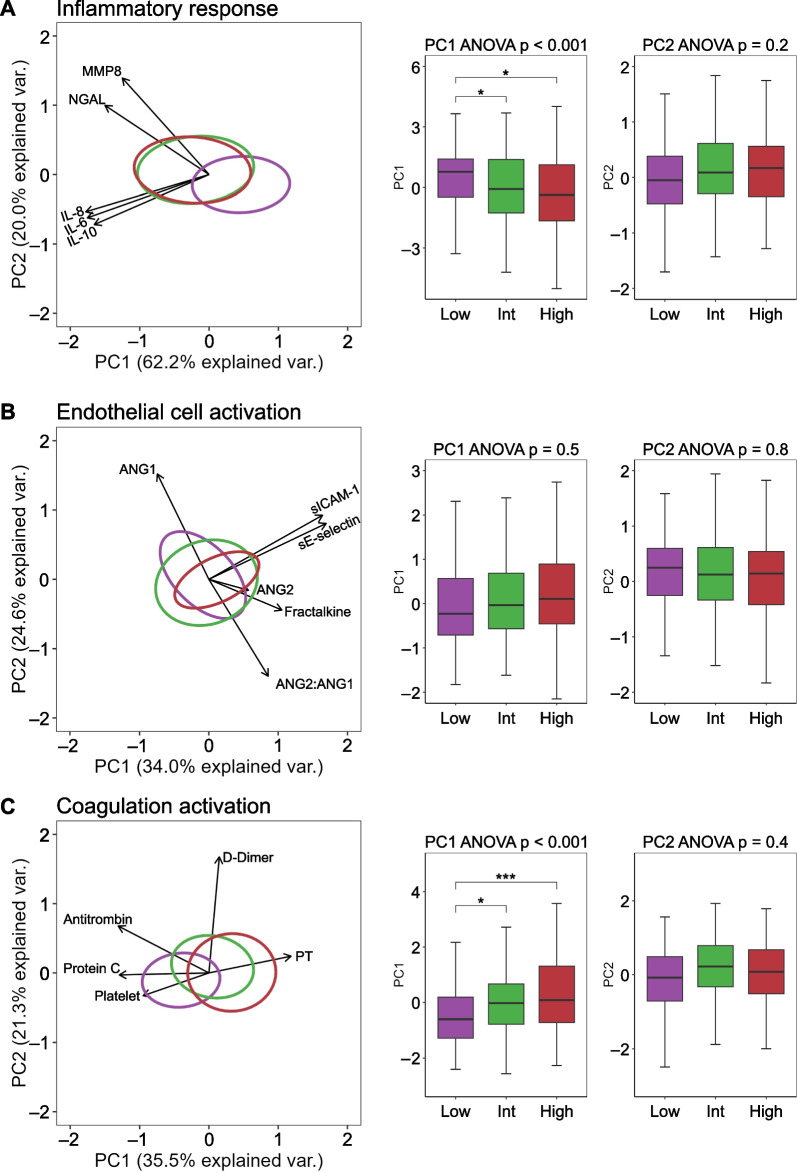
Distinctive and overlapping host response biomarker profiles in plasma among sepsis patients stratified based on MS1 cell percentage in blood. **A** Inflammatory response. **B** Endothelial cell activation and **C** Coagulation activation. Principal component analysis (PCA) in which principal components (PC) 1 and 2 are plotted per pathophysiological domain. Each domain is represented along the *x*- and *y*-axes, labeled with the respective percentage of total variance explained by PC1 and PC2. The contribution of each biomarker to a PC score is detailed in Additional file 1: Table S3. The ellipse illustrates the central 10% of each MS1 group. Arrows in the plot indicate both the direction (arrow orientation) and magnitude (arrow length) of the correlation existing between each biomarker and the PCs. Adjacent to each PCA plot, boxplots facilitate group comparisons concerning PC1 and PC2. It is important to note that even a negative trend within a boxplot of a PC may denote a positive correlation with biomarker concentrations, as reflected by the direction of the arrows. Post-hoc analysis was conducted using a Tukey Test. Significance levels are represented as follows: ***p < 0.001, *p < 0.05. IL interleukin, MMP8 matrix metalloproteinase 8, NGAL neutrophil gelatinase-associated lipocalin, ANG1 angiopoietin 1, ANG2 angiopoietin 2, sE-selectin soluble E-selectin, sICAM-1 soluble intercellular adhesion molecule 1, PT prothrombin time

We determined 15 host response biomarkers reflective of three key pathophysiological domains (inflammatory response, endothelial cell activation, and coagulation activation) in plasma obtained on admission to the ICU (for concentrations of individual biomarkers see Additional file 1: Table S7). First, we generated domain-specific PCA plots to compare patient tertiles with varying MS1 percentages (low, intermediate, and high) (Fig. 2). There was substantial overlap in plasma biomarker responses between MS1 groups; significant differences were detected between the low MS1 group versus the intermediate and high MS1 groups with regard to the PC1 of the inflammatory response (Fig. 2A) and coagulation domains (Fig. 2B). PCA of the endothelial cell response did not reveal differences between MS1 groups (Fig. 2C). The complete contribution of each biomarker to a PC score is depicted in Additional file 1: Table S8. Additional file 1: Fig. S6 presents the magnitude of individual biomarker differences in the low MS1 group relative to the MS1 other groups, expressed as Hedges’ g. This analysis showed that most biomarkers reflective of the inflammatory response and endothelial activation were lower in patients categorized within the low MS1 group, while coagulation activation markers demonstrated a mixed pattern. Notably, these results showed to be robust in the two-group comparison (low vs. high MS1 cell percentage) derived from the k-means clustering approach (Additional file 1: Fig. S7). Taken together, these findings suggest that the relative abundance of MS1 cells has a modest influence on plasma biomarker profiles indicative of pathophysiological pathways implicated in sepsis pathogenesis.

### Relation between MS1 cell proportions and previously described molecular subtypes and signatures in patients with sepsis

**Fig. 3:**
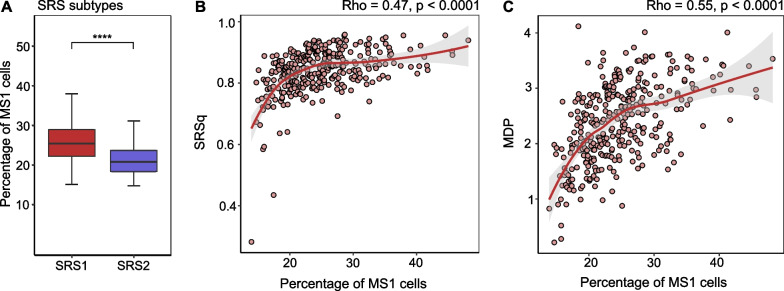
Relation between MS1 cell proportions and previously described molecular subtypes and signatures in patients with sepsis. **A** Percentage of MS1 cells in Sepsis Response Signature (SRS) subtypes. Correlation analysis between the percentage of MS1 cells and **B** quantitative sepsis response signature (SRSq) score, and **C** Molecular Degree of Perturbation (MDP) score. ****Mann–Whitney U test *p* < 0.0001. *rho* spearman correlation coefficient

In the recent past, patients with sepsis have been divided into several subtypes based on blood RNA expression profiles, including Mars1 to Mars4 [17], subtypes named “inflammopathic, “adaptive” and “coagulopathic” [18], and SRS1 and SRS2 [19]. We observed distinct patterns in the distribution of MS1 cell proportions across these subtypes (Fig. 3A and Additional file 1: Fig. S8A, B). Patients classified as the Mars3 subtype—characterized by upregulated adaptive immunity and T cell function and associated with the lowest mortality risk [17]—displayed the lowest percentage of MS1 cells. Likewise, patients within the “adaptive” subtype, characterized by adaptive immune activation and lower mortality [18], also exhibited a lower percentage of MS1 cells. With regard to SRS subtypes, the percentage of MS1 cells was higher in patients of the SRS1 subtype when compared with the SRS2 subtype. The SRS1 subtype entails an immunocompromised profile, encompassing attributes of endotoxin tolerance, T cell exhaustion, downregulation of HLA class II genes, and increased risk of death [19, 20]. Beyond blood RNA profile-based subtypes, patients with ARDS and/or sepsis have also been stratified into hyperinflammatory and hypoinflammatory subtypes [21, 22]. MS1 cells were more abundant in the hyperinflammatory subtype (Additional file 1: Fig. S8C); this subtype is characterized by stronger inflammatory responses, higher mortality rates, and a higher incidence of shock [21].

Based on the SRS subtyping, a quantitative continuous score named SRSq has been generated [20]. SRSq is a score between 0 and 1, with lower values reflecting transcriptomes closer to health and higher values indicating the most severe immune dysregulation. The proportion of MS1 cells showed a moderate positive correlation with SRSq (rho = 0.47, *p* < 0.0001; Fig. 3B). Similarly, the MDP score—which quantifies transcriptional perturbation [23–25]—exhibited a positive correlation with the percentage of MS1 cells (rho = 0.55, *p* < 0.0001; Fig. 3C). Based on the “inflammopathic, “adaptive” and “coagulopathic” subtyping, a continuous probability score has been generated [18]; patients with a high probability score for a given subtype are more likely to belong to that group. Interestingly, MS1 levels showed a positive correlation with the probability scores of the inflammopathic (rho = 0.41, *p* < 0.001; Additional file 1: Fig. S8D) and coagulopathic (rho = 0.22, *p* < 0.01; Additional file 1: Fig. S8E) subtypes, which are characterized by a higher disease severity and mortality [18], and a negative correlation with the probability score of the adaptive phenotype (rho = − 0.42, *p* = 0.0001; Additional file 1: Fig. S8F). MS1 cell percentages also showed a negative correlation with the mean expression levels of HLA class II genes (rho = − 0.48, *p* = 0.0001; Additional file 1: Fig. S8G). HLA class II molecules play a crucial role in driving adaptive immune responses by presenting pathogen-derived peptides to CD4^+^ T cells [26]. Collectively, these results suggest that a higher abundance of MS1 cells is associated with more severe immune dysregulation and immunosuppression.

### MS1 cell proportions in non-infected critically ill patients

**Fig. 4:**
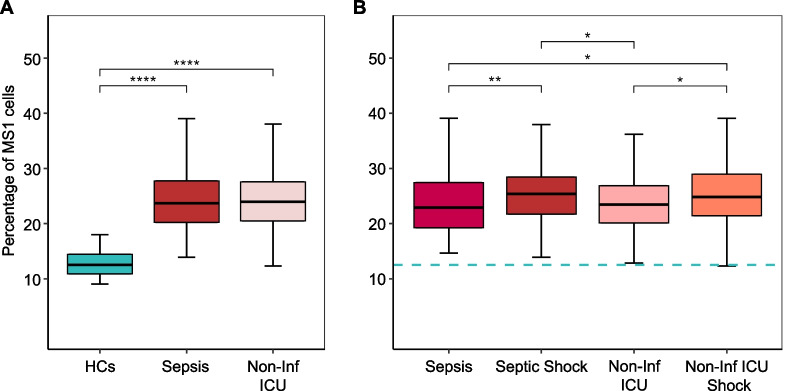
MS1 cell proportions in sepsis patients relative to non-infected critically ill patients. **A** Comparative analysis of MS1 cell levels in healthy controls (HCs, *n* = 42), critically ill patients with infection (Sepsis, *n* = 332) and critically ill patients without infection (Non-Inf ICU, *n* = 215), and, **B** Comparative analysis of MS1 cell levels in patients with sepsis without shock (Sepsis, *n* = 208), patients with sepsis and shock (Septic Shock, *n* = 124), critically ill patients without infection and without shock (Non-Inf ICU, *n* = 135), critically ill patients without infection yet with shock (Non-Inf ICU Shock, *n* = 80). Statistical analyses were performed using the Kruskal–Wallis test with Dunn’s Post-Hoc Test corrected by Benjamini–Hochberg method. ****Dunn’s post-hoc test *p* < 0.0001, **p < 0.01, *p < 0.05

We next compared the percentage of MS1 cells in critically ill patients with sepsis to those in 42 healthy controls and 215 critically ill patients without infection (Fig. 4 and Additional file 1: Table S9 for the comparison of non-infected and sepsis patients). Both groups of critically ill patients displayed elevated MS1 cell proportions in comparison to healthy controls. However, a direct comparison between critically ill patients with sepsis and those with non-infectious conditions did not reveal a statistically significant difference in MS1 cell proportions (Fig. 4A). Notably, in both patient groups, the presence of shock was associated with higher MS1 cell proportions (Fig. 4B).

In an approach similar to our primary analysis, non-infected critically ill patients were classified into tertiles based on their relative MS1 cell abundance: low, intermediate, and high. The high MS1 group exhibited elevated SOFA scores upon ICU admission and higher mortality frequencies (up to day 90) compared to the low MS1 group (Additional file 1: Table S10). Admission diagnoses did not differ between MS1 groups (Additional file 1: Table S11). In a sensitivity analysis also done in sepsis patients, we employed one-dimensional k-means clustering to stratify non-infected critically ill patients based on MS1 cell abundance. Two clusters were defined as the ideal number of clusters (Additional file 1: Table S12). Cluster 2 (high MS1 cell abundance) exhibited elevated SOFA, APACHE IV and APS scores (Additional file 1: Table S13), largely reproducing the results of the comparison between MS1 tertiles.

**Fig. 5:**
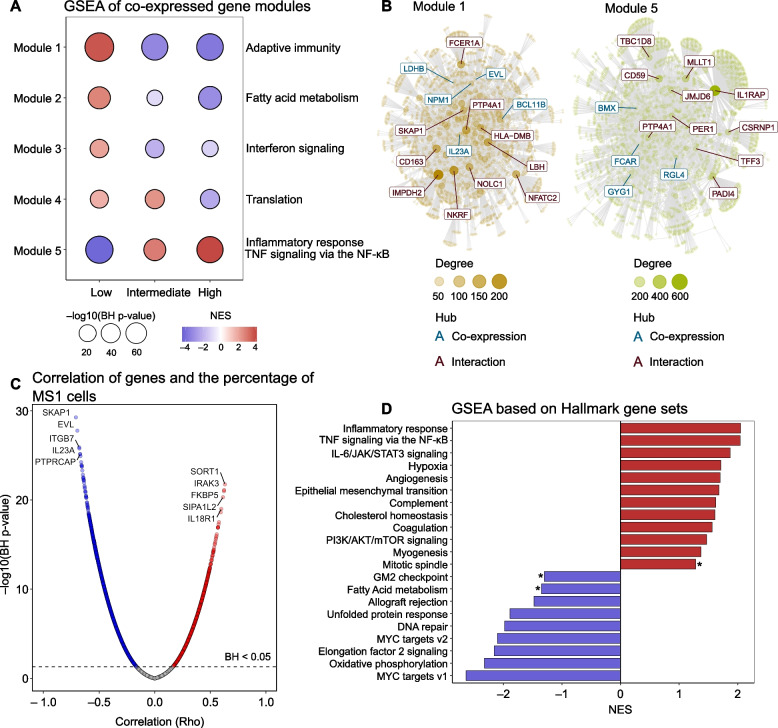
Co-expressed gene modules and gene set enrichment analysis in non-infected critically ill patients stratified according to MS1 cell percentages in blood. **A** The co-expression module identification analysis revealed distinct gene modules based on MS1 cell levels in non-infected critically ill patients. Patients were categorized into low MS1 (*n* = 72), intermediate MS1 (*n* = 71), and high MS1 groups (*n* = 71). The size of each circle in the graph is proportional to -log10(BH adjusted *p* value), and the color represents the normalized enrichment score (NES). Blue indicates a decreased NES, and red color represents an increased NES; **B** The network diagrams depict the two largest differential co-expression gene modules (Module 1 and Module 5) identified in the analysis, the network highlights the hub genes, which are crucial regulatory genes within each module; **C** The graph illustrates the correlation between the percentage of MS1 cells and the normalized gene expression matrix. Positive correlations with a BH adjusted *p* value < 0.05 are shown in red, negative correlations in blue; non-significant correlations are depicted in grey; **D** Gene set enrichment analysis performed on genes that correlated with MS1 levels. The color-coded NES values signify the enrichment score for each pathway; all pathways displayed a BH adjusted *p* value < 0.05, except for pathways marked by an asterisk (*), which indicates a BH adjusted *p* value < 0.1

Furthermore, a gene co-expression network analysis was conducted, first comparing the MS1 tertiles, revealing eight distinct gene expression modules, of which five modules (Fig. 5A) were significantly different between the three groups, with different pathways overrepresented (Additional file 1: Table S14). Modules 1 and 5 exhibited similar expression patterns and pathway enrichments comparable to those seen in Modules 1 and 3 observed in patients with sepsis. Notably, they shared central hub genes, including lymphocyte-related genes like *IL23A*, *SKAP1*, *IMPDH2*, and *NFATC2* (Fig. 5B). A significant overlap between sepsis patients (Fig. 1C, D) and non-infected ICU patients (Fig. 5C, D) was also identified in the functional enrichment analysis based on correlation values (rho) within the gene expression matrix, revealing 15 common pathways, such as EF-2 signaling, allograft rejection, oxidative phosphorylation, and fatty acid metabolism, displayed negative NES, while pathways like inflammatory response, TNF signaling via NF-κB, IL-6/JAK/STAT3 signaling, and hypoxia exhibited increased NES. Interestingly, the inflammatory response (higher in high MS1 group) and adaptive immunity pathways (higher in low MS1 group) were enriched regardless of how patients were stratified (tertiles vs. k-means clustering; Additional file 1: Table S15 and Fig. S9), reproducing findings in patients with sepsis (Additional file 1: Fig. S5). This suggests that the expression of genes related to these pathways is associated with the percentage of MS1 cells irrespective of the presence of infection.

**Fig. 6:**
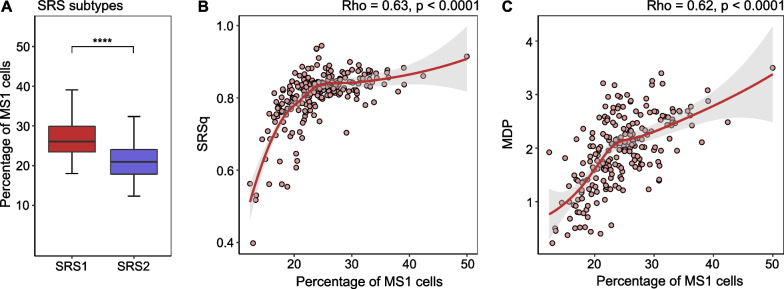
Relation between MS1 cell proportions and previously described molecular subtypes and signatures in critically ill patients without infection. **A** Percentage of MS1 cells in Sepsis Response Signature (SRS) subtypes. Correlation analysis between the percentage of MS1 cells and **B** quantitative sepsis response signature (SRSq) score, and **C** Molecular Degree of Perturbation (MDP) score. ****Mann–Whitney *U* test *p* < 0.0001. *rho* Spearman correlation coefficient

When classifying critically ill patients without infection according SRS subtypes, the percentage of MS1 cells was higher in patients of the SRS1 subtype compared to the SRS2 subtype (Fig. 6A). The proportion of MS1 cells showed a strong positive correlation with SRSq (rho = 0.63, *p* < 0.0001; Fig. 6B). Similarly, the MDP score exhibited a positive correlation with the percentage of MS1 cells (rho = 0.62, *p* < 0.0001; Fig. 6C). Overall, these two scores demonstrated stronger correlations with MS1 cell abundance in critically ill patients without infection compared to those in patients with sepsis.

## Discussion

MDSCs are immature myeloid cells with immunosuppressive features found in increased numbers in the circulation of patients with inflammatory conditions. Expansion of MDSCs is considered to play a key role in sepsis-induced immunosuppression [5]. Two recent studies reported a newly discovered monocyte state named MS1, reminiscent of M-MDSCs, of which the abundance in blood of patients with sepsis correlated with higher mortality rates [7, 8]. The present study provides comprehensive information about the association between the proportion of MS1 cells, and disease presentation, complications and host response aberrations in critically ill patients with sepsis or a non-infectious condition.

Previously, expression of the MS1 gene program in blood was reported to be negatively associated with survival in an analysis making use of 11–15 cohorts included in meta-analyses reporting on mortality among sepsis patients [7, 8]. In our study, MS1 cell abundance did not differ between sepsis survivors and non-survivors, and there were no mortality differences between patient groups stratified according to MS1 cell frequencies. Albeit non-significant, MS1 cell percentages expressed as a continuous variable showed a nonlinear relationship with mortality. While this might indicate that an increase in MS1 cell abundance to a certain extent may improve outcome, this possibility is speculative and requires validation. Notably, in the earlier meta-analyses the association between the MS1 cell abundance and mortality was not consistent in all individual sepsis cohorts [7, 8].

Thus far, the relation between MS1 cell frequency, and the clinical presentation and disease associated complications in patients with sepsis was not studied in great detail. We found a clear association between the percentage of MS1 cells and disease severity, as evidenced by higher severity scores and more shock in the intermediate and high MS1 groups. Additionally, the high MS1 group more often presented with abdominal infection, which however most likely was linked to the fact that these patients more often had shock on admission to the ICU, more so than that the MS1 expansion was related to the site of infection. In agreement, we and others previously reported a higher incidence of shock in sepsis patients with an abdominal source of infection [13, 27]. MS1 cells clearly exert immune suppressive effects, and accordingly, the proportion of MS1 cells in sepsis patients displayed a negative correlation with HLA class II gene expression [7, 8] (considered a classic sign of immunosuppression) [28]. Nevertheless, the MS1 score did not differ between patients who did and those who did not develop a secondary infection, a complication considered to be linked with immunosuppression [28]. In contrast, patients with high MS1 cell abundance more often developed ARDS while on the ICU, a complication that is considered to arise from exaggerated inflammation [29]. These data suggest that, in spite of the immune suppressive properties of MS1 cells [7, 8], other concurrent host response aberrations may be dominant in the overall immune state of sepsis patients with high MS1 cell proportions. Indeed, our comprehensive host response analyses provide support for this notion.

We studied the association between MS1 cell abundance and the host response by analyzing blood gene expression profiles and, in a more targeted way, by evaluating 15 plasma biomarkers reflective of pathophysiological mechanisms implicated in sepsis. We applied different gene set enrichment techniques to show that an increase in MS1 cell frequency is associated with a decrease in lymphocyte-related and interferon response genes. These results support the conclusions by Reyes et al. [8], highlighting a reduced interferon response in MS1 cells upon stimulation and a negative correlation between the MS1 gene program and expression of interferon response genes, and are consistent with the recognized immune suppressive activity of MDSCs, necessitating the inactivation of the interferon pathway [30]. On the other hand, an increased percentage of MS1 cells was related to upregulation of TNF signaling pathways via NF-κB, IL-6/JAK/STAT3 signaling, and inflammatory response pathways. These seemingly paradoxical findings fit with the current consensus that hyperinflammation and immune suppression co-exist in sepsis patients upon ICU admission [4], rather than that they represent successive phases [31]. Plasma biomarker analysis demonstrated only modest differences in the inflammation domain (lower in patients with low MS1 cell numbers), while endothelial and coagulation responses were similar across MS1 groups.

Recent studies have tried to stratify patients with sepsis into more homogeneous subgroups based on host response characteristics using various unsupervised clustering methods [17–19, 21, 22]. We grouped patients included in our cohort in these previously published subtypes and determined the MS1 cell frequency in each subgroup, thereby seeking to assess potential overlap. Patients with low MS1 cell percentages more often classified in the low mortality risk subtypes Mars3 [17], adaptive [18], and hypoinflammatory [21, 22], which is in agreement with the association between MS1 cell frequency and disease severity. Otherwise, MS1 cell proportions did not clearly align with dominant pathophysiological mechanisms; for example, higher MS1 cell abundances were found in the SRS1 subtype (which mainly reflects an immunocompromised profile) [19, 20], but also in the hyperinflammatory subtype (reflecting a subtype with dominant inflammatory host response pattern); these seemingly opposing associations are in agreement with our gene set enrichment analyses discussed above. We found positive correlations between MS1 cell abundance and the SRSq [19, 20] and MDP scores, which indicate the extent of gene expression perturbation relative to a healthy state [23–25]. Concurrently, the proportion of MS1 cells had a negative correlation with HLA class II gene expression, corroborating earlier findings [7, 8] and pointing at immunosuppression [28]. Collectively, these results suggest that, while MS1 cells clearly exert immune suppressive effects [7, 8], their abundance in patients with sepsis upon ICU admission should be considered as one aberrant feature in a broadly disturbed host response in patients who already are critically ill (i.e., have departed from normal immune homeostasis along divergent pathophysiological pathways).

A previous study reported that the MS1 cell fraction is also expanded in ICU patients with a non-infectious condition, although to a lesser extent than in sepsis patients admitted to the ICU [7]. Contrary to these findings, our study found no difference between sepsis patients and non-infected ICU controls, and we further show that the presence of shock is similarly associated with MS1 cell expansion in both groups. The discrepancy between our study and the one published earlier may be explained by differences in disease severities between sepsis and non-infected patients (not reported in [7]). Analyses seeking to associate MS1 cell frequencies with other host response deviations in non-infected ICU patients showed strong similarities with results obtained in sepsis patients, including a decrease in lymphocyte-related and interferon response genes, and an upregulation of TNF signaling pathways via NF-κB, IL-6/JAK/STAT3 signaling, and inflammatory response pathways in patients with higher MS1 cell fractions. Together, these data suggest that MS1 cell expansion and its relation to other host response aberrations are primarily determined by the severity of disease and not by the inciting injury, thereby aligning with the recently proposed new concept of critical illness [32].

Our study has strengths and limitations. We used a large well-annotated cohort of prospectively enrolled patients, allowing studies in ICU patients with or without sepsis on clinically relevant outcomes and associations with diverse host response mechanisms. The study was conducted in two ICUs in the Netherlands; results may not be generalizable to other critical care settings. Our investigation was observational and therefore does not allow conclusions about causal relationships. CIBERSORTx can only estimate cell abundances based on the cells represented in the reference matrix. Therefore, the percentages estimated using whole blood RNA profiles in this and previously reported studies [7, 8] refer to the subpopulation of cells within the peripheral blood mononuclear cell fraction. Another limitation lies in the inherent nature of deconvolution, which does not provide a definitive picture of cellular composition. Although implementing scRNA-seq analyses and/or mass cytometry could offer more direct information to identify MS1 cells and confirm or refute the hypotheses advanced in this paper, applying these technologies in cohort studies of this magnitude poses financial and logistical challenges.

## Conclusions

This study establishes a prominent association between elevated MS1 cell percentages, and increased disease severity and shock in patients admitted to the ICU with sepsis or a non-infectious condition. The association between MS1 cell abundance and diverse host response anomalies highlights its potential as a indicator of broad immune dysregulation, entailing not only immune suppression but also perturbations signifying exaggerated inflammation.

### Supplementary Information

**Additional file 1.** Supplementary materials, tables and figures.

## Data Availability

The MARS cohort is available at the Gene Expression Omnibus public repository of NCBI under accession number GSE65682. This study did not generate an original code. All essential codes used in our study are publicly available on GitHub: https://github.com/GiuseppeLeite/ms1_mars**.**

## Abbreviations

AKI: Acute kidney injury
APACHE IV APS: Acute physiology and chronic health evaluation IV acute physiology
ARDS: Acute respiratory distress syndrome
BH correction: Benjamini–Hochberg correction
HLA: Human leucocyte antigen
IL: Interleukin
MDSCs: Myeloid-derived suppressor cells
MHC-II: Class II major histocompatibility complex
M-MDSCs: Monocytic myeloid-derived suppressor cells
MS1: Monocyte state 1
NES: Normalized enrichment scores
NGAL: Neutrophil gelatinase-associated lipocalin
NK: Natural killer
PCA: Principal component analysis
PMN-MDSCs: Polymorphonuclear myeloid-derived suppressor cells
rho: Spearman correlation coefficients
sE-selectin: Soluble E-selectin
SOFA: Sequential organ failure assessment
SRSq: Quantitative sepsis response signature
