# Supplementary material for: Monocyte state 1 (MS1) cells in critically ill patients with sepsis or non-infectious conditions: association with disease course and host response
Source: Crit Care. 2024 Mar 19;28:88. doi: 10.1186/s13054-024-04868-5 (PMC10953179; doi:10.1186/s13054-024-04868-5)
Supplement: Supplementary file 1 — Additional file 1. Supplementary materials, tables and figures. [file 13054_2024_4868_MOESM1_ESM.docx]

**Supplementary material**

**Monocyte state 1 (MS1) cells in critically ill patients with sepsis or non-infectious conditions: association with disease course and host response**

Giuseppe G.F. Leite^1,2^, Justin de Brabander^1^, Erik H.A. Michels^1^, Joe M. Butler^1^, Olaf L. Cremer^3^, Brendon P. Scicluna^1,4,5^, Timothy E. Sweeney^6^, Miguel Reyes^7^, Reinaldo Salomao^2^, Hessel Peters-Sengers^1,8^, and Tom van der Poll^1,9^

**Overview of Content**

**Supplementary Methods3**

Definitions3

*Comorbidities3*

*Organ dysfunctions3*

*Intensive care unit (ICU)-acquired complications4*

Measurements4

*Blood gene expression microarrays4*

*Plasma biomarker measurements5*

Bioinformatics analyses6

*Gene co-expression network and module6*

*Correlation between the percentage of MS1 cells and the gene expression matrix6*

*Correlation analysis between the percentage of MS1 cells and other molecular signatures6*

**Supplementary Tables9**

**Table S19**

**Table S210**

**Table S311**

**Table S412**

**Table S513**

**Table S615**

**Table S717**

**Table S818**

**Table S919**

**Table S1020**

**Table S1121**

**Table S1222**

**Table S1323**

**Table S1424**

**Table S1526**

**Supplementary Figures28**

**Figure S128**

**Figure S229**

**Figure S330**

**Figure S431**

**Figure S532**

**Figure S633**

**Figure S734**

**Figure S836**

**Figure S938**

**References39**

**Supplementary Methods**

Definitions

*Comorbidities*

Cardiovascular compromise was defined as a medical history of congestive heart failure, chronic cardiovascular disease, myocardial infarction, peripheral vascular disease or cerebrovascular disease. Malignancy was defined as a medical history of either metastatic or not metastatic solid tumor, or hemodynamic malignancy. Renal insufficiency was defined as a history of chronic renal insufficiency, or treatment with chronic intermittent hemodialysis or continuous ambulatory peritoneal dialysis. Respiratory insufficiency was defined as a history of chronic respiratory insufficiency, chronic obstructive pulmonary disease, or treatment at home with oxygen or ventilator support. Immune compromise was defined as a history of immune deficiency, human immunodeficiency virus (HIV) infection, acquired immune deficiency syndrome (AIDS), asplenia, or chronic use of corticosteroids, antineoplastic or other immune suppressive medications. Chronic comorbid conditions were scored using the Charlson comorbidity index [1].

*Organ dysfunctions*

Sepsis was defined according to the Sepsis 3.0 definition as the presence of an infection and organ dysfunction(s) represented by two or more Sequential Organ Failure Assessment (SOFA) points [2]. When the SOFA score was not available (5%), organ failure was defined by presence of mechanical ventilation, acute kidney injury (AKI), acute respiratory distress syndrome (ARDS) or shock. Shock was defined by the use of vasopressors (norepinephrine, epinephrine or dopamine) for hypotension in a norepinephrine-equivalent dose of more than 0.1 µg/kg/min. ARDS was prospectively defined using strict preset criteria [3]. AKI was prospectively defined according to the risk, injury, failure, loss, and end-stage kidney disease (RIFLE) classification and the Kidney Disease: Improving Global Outcomes (KDIGO) criteria [4, 5].

*Intensive care unit (ICU)-acquired complications*

ICU-acquired infection was defined as any new-onset infection starting more than 48 hours after ICU admittance, and for which the attending physician started a new antibiotic regimen. ICU-acquired ARDS was defined as ARDS diagnosed more than 48 hours after ICU admission.

Measurements

*Blood gene expression microarrays*

Whole blood was drawn in PAXgene™ tubes (Becton– Dickinson, Breda, the Netherlands) within 24 h after ICU admission. PAXgene™ blood samples were also collected from 42 healthy controls [median age 35 years (interquartile range 30–63); 57% male] after obtaining written informed consent. Total RNA was extracted using the PAXgene blood mRNA kit (Qiagen, Venlo, the Netherlands), according to manufacturer’s instructions. Total RNA (RNA integrity number > 6.0) was processed and hybridized to the Affymetrix Human Genome U219 96-array and scanned by using the GeneTitan instrument at the Cologne Center for Genomics (CCG, Cologne, Germany), as described by the manufacturer (Affymetrix). Raw data scans (.CEL files) were read into the R language and environment for statistical computing (version 2.15.1; R Foundation for Statistical Computing, Vienna, Austria; http://www.R-project.org/). Gene expression data sets are available at the Gene Expression Omnibus public repository of NCBI under the accession number GSE65682. Pre-processing and quality control were performed by using the Affy package version 1.36.1. Array data were background corrected by robust multi-array average, quantiles-normalized and summarized by median polish using the expresso function. The occurrence of non-experimental chip-effects was evaluated by means of the Surrogate Variable Analysis (R package version 3.4.0) and corrected by the empirical Bayes Method ComBat [6, 7]. The 49,386 log-transformed probes were mapped to the corresponding genes using GPL13667, probes with more than one corresponding gene were removed, for duplicate probes for the same gene symbol, the probe with the highest overall expression value was retained.

*Plasma biomarker measurements*

Biomarkers indicative of host response pathways implicated in sepsis pathogenesis were measured on admission to the ICU in the subset of patients with sepsis. EDTA anticoagulated blood was collected on admission and stored within 4 hours at -80 °C until use. Interleukin (IL)-6, IL-8, IL-10, soluble E-selectin, soluble intercellular adhesion molecule-1 (sICAM-1) and fractalkine were measured by FlexSet cytometric bead array (BD Biosciences, San Jose, CA) using FACSCalibur flow cytometer (Becton Dickenson, Franklin Lakes, NJ). Matrix metalloproteinase-8 (MMP-8), NGAL**,** angiopoietin-1, angiopoietin-2, protein C, antithrombin, (all R&D systems, Abingdon, UK) and D-dimer (Procartaplex, eBioscience, San Diego, CA) were measured by Luminex multiplex assay using BioPlex 200 (BioRad, Hercules, CA). Prothrombin time (PT) was determined by using a photometric method with Dade Innovin Reagent (Siemens Healthcare Diagnostics).

Bioinformatics analyses

*Gene co-expression network and module analysis*

Weighted gene co-expression network analysis (WGCNA)-based modular analysis was done on the microarray data comparing the three groups using the R-based Co-Expression Modules identification Tool (CEMiTool) package [8]. The co-expressed modules were subjected to overrepresentation analysis using the Molecular Signatures Database (MSigDB) with pathway datasets listed in hallmark gene sets and Reactome [9]. Relevant modules were identified based on significant differences in the module eigengene between patient groups (assessed using Mann-Whitney U or Kruskal-Wallis tests) and the presence of significantly overrepresented pathways (FDR < 0.05).

*Correlation between the percentage of MS1 cells and the gene expression matrix*

Based on the normalized gene expression matrix, we performed the correlation analysis of their expression with the percentage of MS1 cells. Spearman correlation coefficients (rho). We ranked all genes based on rho values and performed gene set enrichment analysis using the “fgsea” package with the hallmark gene sets.

*Correlation analysis between the percentage of MS1 cells and other molecular signatures*

We performed correlation analyses to examine the relationship between the percentage of MS1 cells and previously described molecular signatures. Spearman's rank correlation coefficient (rho) was used to assess the strength and direction of these correlations, with statistical significance determined by BH-adjusted p-values < 0.05. The molecular signatures examined in the correlation analysis encompassed the following:

1. Quantitative sepsis response signature (SRSq) score [10] – The SRSq score was computed using a 19-gene set as the extended signature (including genes: *SLC25A38*, *DNAJA3*, *NAT10*, *THOC1*, *MRPS9*, *PGS1*, *UBAP1*, *USP5*, *TTC3*, *SH3GLB1*, *BMS1*, *FBXO31*, *ARL14EP*, *CCNB1IP1*, *DYRK2*, *ADGRE3*, *MDC1*, *TDRD9*, and *ZAP70*). This SRSq score ranges from 0 to 1, with values close to zero indicating patients are likely to be healthy, while values near one signify a high-risk status.
2. Molecular degree of perturbation (MDP) score [11] – which functions as a quantitative representation of transcriptional perturbation. This score is derived by computing the average of the highest 25% gene z-scores related to expression levels between patients with sepsis and the reference group ("healthy controls").
3. Human leucocyte antigen (HLA) class II gene mean expression (including *HLA-DRA, HLA-DPA1, HLA-DRB1, HLA-DPB1, HLA-DMB, HLA-DQA1, HLA-DOA, HLA-DRB1, HLA-DOB, HLA-DQA2* and *HLA-DPA3*). This score was computed as the mean expression of HLA genes present in our gene expression matrix.
4. Sweeney subtypes probabilities were calculated as previously described [12]. In brief, each of the 33 mRNAs is assigned to one of three groups, and we calculated the difference of geometric means of gene expression for each grouping. The groupings are as follows: Inflammopathic (*ARG1, LCN2, LTF, OLFM4, HLA-DMB)*; Adaptive (*YKT6, PDE4B, TWISTNB, BTN2A2, ZBTB33, PSMB9, CAMK4, TMEM19, SLC12A7, TP53BP1, PLEKHO1, SLC25A22, FRS2, GADD45A, CD24, S100A12, STX1A)*; and Coagulopathic (*KCNMB4, CRISP2, HTRA1, PPL, RHBDF2, ZCCHC4, YKT6, DDX6, SENP5, RAPGEF1, DTX2, RELB)*. We then applied the previously defined multi-class logistic regression model to these three input gene expression scores, which yields a probability of endotype assignment (for each subject, the total probability [p(Inflammopathic) + p(Adaptive) + p(Coagulopathic)] sums to 1). Each sample is assigned a subtype according to the highest probability

Furthermore, we investigated the SRS1 and SRS2 subtypes [13]; the inflammopathic, adaptive and coagulopathic subtypes [12]; the Mars subtypes described by our group [14]; and the hyperinflammatory and hypoinflammatory subtypes, originally identified in patients with acute respiratory distress syndrome (ARDS) based on three plasma biomarkers (IL-8, bicarbonate, and protein C) [15] and recently also documented in patients with sepsis [16]. The SRSq score and the MDP score were also assessed in critically ill patients without infection.

**Supplementary Tables**

**Table S1. Baseline characteristics and outcomes of patients admitted to the ICU with sepsis stratified based on the presence of abdominal or respiratory infections**

| **Characteristic** | **Abdominal**, (N = 70)*^1^* | **Respiratory**, (N = 122)*^1^* | **p-value** |
| --- | --- | --- | --- |
| Percentage of MS1 cells | 26.1 (22.5, 29.5) | 23.8 (19.6, 27.9) | **0.01** |
| **Demographics** | | | |
| Age years | 64.0 (56.0, 71.0) | 65.0 (52.8, 73.8) | 0.9 |
| White race | 61 (87%) | 100 (82%) | 0.5 |
| Male sex | 35 (50%) | 80 (66%) | 0.05 |
| Admission type, surgery | 32 (46%) | 13 (11%) | **<0.00001** |
| BMI | 25.4 (22.8, 30.2) | 24.2 (21.7, 26.6) | 0.05 |
| **Comorbidity** | | | |
| Charlson score | 4.0 (3.0, 5.0) | 4.0 (2.0, 6.0) | >0.9 |
| Cardiovascular insufficiency | 3 (4.3%) | 4 (3.3%) | >0.9 |
| Respiratory insufficiency | 3 (4.3%) | 14 (11%) | 0.2 |
| Renal insufficiency | 8 (11%) | 9 (7.4%) | 0.5 |
| Hypertension | 21 (30%) | 24 (20%) | 0.1 |
| Diabetes mellitus | 13 (19%) | 16 (13%) | 0.4 |
| COPD | 7 (10%) | 30 (25%) | 0.02 |
| Cerebrovascular disease | 3 (4.3%) | 16 (13%) | 0.09 |
| **Disease severity on admission** | | | |
| SOFA Score | 7.5 (6.0, 10.0) | 6.0 (4.0, 8.0) | **0.001** |
| APACHE IV Score | 79.5 (63.0, 93.5) | 73.0 (62.0, 95.0) | 0.7 |
| APS | 65.5 (51.5, 84.0) | 63.5 (49.3, 81.8) | 0.7 |
| ARDS | 16 (23%) | 42 (34%) | 0.1 |
| AKI | 30 (43%) | 25 (20%) | **0.001** |
| Shock | 31 (44%) | 33 (27%) | **0.02** |
| **Outcomes** | | | |
| Hospital LOS, days | 22.0 (8.5, 53.5) | 13.5 (7.0, 28.8) | 0.04 |
| ICU LOS, days | 3.0 (1.3, 8.8) | 7.0 (3.0, 11.0) | **0.008** |
| **ICU‑acquired complications** | | | |
| ARDS | 9 (13%) | 17 (14%) | >0.9 |
| Infection | 7 (10%) | 13 (11%) | >0.9 |
| **Mortality** | | | |
| Death in ICU | 12 (17%) | 22 (18%) | >0.9 |
| 30‐day mortality | 16 (23%) | 34 (28%) | 0.6 |
| 60‐day mortality | 19 (27%) | 39 (32%) | 0.6 |
| 90‐day mortality | 22 (31%) | 40 (33%) | >0.9 |

^1^ Median (IQR); n / N (%)

Abbreviations: AKI: acute kidney injury, APACHE: acute physiology and chronic health evaluation, APS: acute physiology scores, ARDS: acute respiratory distress syndrome, BMI: body mass index, COPD: chronic obstructive pulmonary disease, LOS: length of stay, SOFA: sequential organ failure assessment

**Table S2. Baseline characteristics and outcomes of patients admitted to the ICU with septic shock stratified based on the presence of abdominal or respiratory infections**

| **Characteristic** | **Abdominal shock** (N = 31)*^1^* | **Respiratory shock** (N = 33)*^1^* | **p-value** |
| --- | --- | --- | --- |
| MS1 relative percentage | 26.1 (24.7, 28.1) | 26.2 (21.4, 30.0) | 0.95 |
| **Demographics** | | | |
| Age years | 66.0 (58.5, 71.0) | 64.0 (55.0, 71.0) | 0.7 |
| White race | 29 (94%) | 26 (79%) | 0.2 |
| Male sex | 15 (48%) | 24 (73%) | 0.08 |
| Admission type, surgery | 15 (48%) | 7 (21%) | **0.04** |
| BMI | 25.2 (23.0, 30.3) | 24.7 (23.2, 26.3) | 0.2 |
| **Comorbidity** | | | |
| Charlson score | 4.0 (3.0, 5.5) | 4.0 (3.0, 6.0) | 0.9 |
| Cardiovascular insufficiency | 2 (6.5%) | 2 (6.1%) | >0.9 |
| Respiratory insufficiency | 3 (9.7%) | 3 (9.1%) | >0.9 |
| Renal insufficiency | 5 (16%) | 4 (12%) | 0.9 |
| Hypertension | 11 (35%) | 7 (21%) | 0.3 |
| Diabetes mellitus | 8 (26%) | 7 (21%) | 0.9 |
| COPD | 6 (19%) | 7 (21%) | >0.9 |
| Cerebrovascular disease | 2 (6.5%) | 2 (6.1%) | >0.9 |
| **Disease severity on admission** | | | |
| SOFA Score | 9.0 (8.0, 12.0) | 9.0 (7.0, 11.0) | 0.2 |
| APACHE IV Score | 91.0 (73.5, 104.5) | 85.0 (64.0, 114.0) | 0.7 |
| APS | 77.0 (62.5, 92.5) | 73.0 (52.0, 101.0) | 0.8 |
| ARDS | 11 (35%) | 19 (58%) | 0.1 |
| AKI | 19 (61%) | 13 (39%) | 0.1 |
| **Outcomes** | | | |
| Hospital LOS, days | 43.0 (9.0, 66.5) | 16.0 (8.0, 46.0) | 0.3 |
| ICU LOS, days | 8.0 (4.5, 18.0) | 9.0 (7.0, 12.0) | 0.7 |
| **ICU‑acquired complications** | | | |
| ARDS | 6 (19%) | 4 (12%) | 0.7 |
| Infection | 5 (16%) | 4 (12%) | 0.91 |
| **Mortality** | | | |
| Death in ICU | 9 (29%) | 9 (27%) | >0.9 |
| 30‐day mortality | 11 (35%) | 11 (33%) | >0.9 |
| 60‐day mortality | 13 (42%) | 12 (36%) | 0.8 |
| 90‐day mortality | 14 (45%) | 12 (36%) | 0.6 |

^1^ Median (IQR); n / N (%)

Abbreviations: AKI: acute kidney injury, APACHE: acute physiology and chronic health evaluation, APS: acute physiology scores, ARDS: acute respiratory distress syndrome, BMI: body mass index, COPD: chronic obstructive pulmonary disease, LOS: length of stay, SOFA: sequential organ failure assessment

**Table S3. Optimal number of clusters based on the percentage of MS1 cells in patients admitted to the ICU with sepsis: consensus-based algorithm results**

| **Number of Clusters** | **Method** | **Package** | **Duration** |
| --- | --- | --- | --- |
| 1 | GapMaechler2012 | easystats | 1.66 |
| 1 | GapDudoit2002 | easystats | 1.73 |
| 2 | Elbow | easystats | 0.18 |
| 2 | Silhouette | easystats | 0.05 |
| 2 | kl | NbClust | 0.09 |
| 2 | Duda | NbClust | 0.02 |
| 2 | Pseudot2 | NbClust | 0.02 |
| 2 | Beale | NbClust | 0.03 |
| 2 | PtBiserial | NbClust | 1.61 |
| 2 | Frey | NbClust | 0.20 |
| 2 | Mcclain | NbClust | 0.18 |
| 2 | Mixture (V) | mclust | 0.29 |
| 2 | Mixture (E) | mclust | 0.29 |
| 3 | Hartigan | NbClust | 0.07 |
| 3 | CCC | NbClust | 0.05 |
| 3 | Scott | NbClust | 0.04 |
| 3 | Tracew | NbClust | 0.04 |
| 3 | Ball | NbClust | 0.03 |
| 4 | Dunn | NbClust | 0.05 |
| 4 | SDindex | NbClust | 0.03 |
| 5 | Ratkowsky | NbClust | 0.03 |
| 8 | Marriot | NbClust | 0.04 |
| 8 | Friedman | NbClust | 0.03 |
| 8 | Rubin | NbClust | 0.03 |
| 9 | DB | NbClust | 0.02 |
| 10 | Ch | NbClust | 0.06 |
| 10 | Cindex | NbClust | 0.05 |
| 10 | SDbw | NbClust | 0.24 |

**Table S4. Baseline characteristics and outcomes of patients admitted to the ICU with sepsis stratified into clusters by percentage of MS1 cells using one-dimensional k-means clustering**

| **Characteristic** | **Cluster 1** (Low, N = 176)*^1^* | **Cluster 2** (High, N = 156)*^1^* | **p-value** |
| --- | --- | --- | --- |
| MS1 relative percentage | 20.4 (18.4, 22.3) | 28.2 (26.1, 32.4) | **<0.00001** |
| **Demographics** | | | |
| Age years | 63.5 (52.5, 73.0) | 65.0 (56.0, 74.0) | 0.31 |
| White race | 142 (81%) | 140 (90%) | **0.03** |
| Male sex | 105 (60%) | 92 (59%) | 0.98 |
| Admission type, surgery | 48 (27%) | 43 (28%) | >0.99 |
| BMI | 24.9 (22.6, 29.2) | 24.8 (22.2, 28.1) | 0.45 |
| **Comorbidity** | | | |
| Charlson score | 4.0 (2.0, 5.0) | 4.0 (3.0, 5.3) | 0.07 |
| Cardiovascular insufficiency | 7 (4.0%) | 9 (5.8%) | 0.61 |
| Respiratory insufficiency | 12 (6.8%) | 12 (7.7%) | 0.92 |
| Renal insufficiency | 24 (14%) | 14 (9.0%) | 0.24 |
| Hypertension | 53 (30%) | 36 (23%) | 0.18 |
| Diabetes mellitus | 31 (18%) | 28 (18%) | >0.99 |
| COPD | 24 (14%) | 25 (16%) | 0.64 |
| Cerebrovascular disease | 15 (8.5%) | 12 (7.7%) | 0.94 |
| **Site of Infection** | | | |
| Respiratory | 64 (36%) | 58 (37%) | 0.96 |
| Abdominal | 26 (15%) | 44 (28%) | **0.004** |
| Cardiovascular | 11 (6.3%) | 5 (3.2%) | 0.3 |
| Urinary | 10 (5.7%) | 14 (9.0%) | 0.34 |
| CNS | 2 (1.1%) | 2 (1.3%) | >0.99 |
| Skin | 9 (5.1%) | 8 (5.1%) | >0.99 |
| Other | 7 (4.0%) | 3 (1.9%) | 0.44 |
| Unknown | 3 (1.7%) | 2 (1.3%) | >0.99 |
| Mix infection | 44 (25%) | 20 (13%) | **0.007** |
| **Disease severity on admission** | | | |
| SOFA Score | 7.0 (5.0, 9.0) | 7.5 (5.0, 10.0) | 0.06 |
| APACHE IV Score | 76.0 (60.8, 93.5) | 82.0 (64.0, 104.0) | **0.04** |
| APS | 63.5 (50.0, 81.0) | 70.0 (52.0, 91.3) | **0.05** |
| ARDS | 41 (23%) | 49 (31%) | 0.12 |
| AKI | 63 (35%) | 64 (41%) | 0.32 |
| Shock | 48 (27%) | 76 (49%) | **<0.00001** |
| **Outcomes** | | | |
| Hospital LOS, days | 15.0 (8.0, 35.0) | 17.0 (8.0, 40.5) | 0.52 |
| ICU LOS, days | 4.5 (2.0, 10.0) | 6.0 (2.0, 11.0) | 0.2 |
| **ICU‑acquired complications** | | | |
| ARDS | 13 (7.4%) | 28 (18%) | **0.005** |
| Infection | 17 (9.7%) | 22 (14%) | 0.27 |
| **Mortality** | | | |
| Death in ICU | 35 (20%) | 31 (20%) | >0.99 |
| 30‐day mortality | 48 (27%) | 45 (29%) | 0.84 |
| 60‐day mortality | 54 (31%) | 55 (35%) | 0.44 |
| 90‐day mortality | 56 (32%) | 61 (39%) | 0.2 |

^1^ Median (IQR); n / N (%)

Abbreviations: AKI: acute kidney injury, APACHE: acute physiology and chronic health evaluation, APS: acute physiology scores, ARDS: acute respiratory distress syndrome, BMI: body mass index, CNS: central nervous system; COPD: chronic obstructive pulmonary disease, LOS: length of stay, SOFA: sequential organ failure assessment

**Table S5**. **Gene module overrepresentation analysis of patients admitted to the ICU with sepsis stratified into tertiles by percentage of MS1 cells**

| **Module 1** | | | |
| --- | --- | --- | --- |
| **Gene Set Name** | **Pathway name** | **k/K** | **FDR** |
| Hallmark | Allograft rejection | 7% | 8.45E-13 |
| Hallmark | IL2/STAT5 signaling | 4% | 1.23E-06 |
| Hallmark | Estrogen response early | 4% | 1.23E-06 |
| Hallmark | Myc targets v1 | 4% | 1.23E-06 |
| Hallmark | Interferon gamma response | 4% | 1.56E-05 |
| Reactome | Adaptive immune system | 2% | 2.86E-10 |
| Reactome | Generation of second messenger molecules | 15% | 6.72E-07 |
| Reactome | TCR signaling | 6% | 9.67E-07 |
| Reactome | MHC class II antigen presentation | 6% | 1.61E-05 |
| Reactome | PD1 signaling | 14% | 2.27E-04 |
| **Module 2** | | | |
| **Gene Set Name** | **Pathway name** | **k/K** | **FDR** |
| Hallmark | Interferon gamma response | 30% | 6.86E-104 |
| Hallmark | Interferon alpha response | 51% | 7.96E-100 |
| Hallmark | Inflammatory response | 6% | 1.72E-11 |
| Hallmark | TNF signalling via NF-κB | 5% | 6.37E-09 |
| Hallmark | Allograft rejection | 4% | 1.63E-06 |
| Reactome | Interferon signaling | 17% | 5.96E-47 |
| Reactome | Interferon alpha beta signaling | 32% | 3.56E-38 |
| Reactome | Cytokine signaling in immune system | 5% | 1.78E-36 |
| Reactome | Interferon gamma signaling | 16% | 1.60E-19 |
| Reactome | Antiviral mechanism by IFN-stimulated genes | 16% | 8.98E-17 |
| **Module 3** | | | |
| **Gene Set Name** | **Pathway name** | **k/K** | **FDR** |
| Hallmark | TNF signalling via NF-κB | 5% | 1.82E-05 |
| Hallmark | Hypoxia | 4% | 9.86E-05 |
| Hallmark | Inflammatory response | 4% | 6.29E-04 |
| Hallmark | Adipogenesis | 3% | 3.63E-03 |
| Hallmark | Cholesterol homeostasis | 5% | 3.63E-03 |
| Reactome | Neutrophil degranulation | 4% | 1.39E-07 |
| Reactome | Innate immune system | 2% | 1.59E-06 |
| Reactome | Metabolism of lipids | 2% | 8.06E-04 |
| Reactome | Signaling by interleukins | 3% | 8.06E-04 |
| Reactome | Metabolic disorders of biological oxidation enzymes | 12% | 5.57E-03 |
| **Module 4** | | | |
| **Gene Set Name** | **Pathway name** | **k/K** | **FDR** |
| Hallmark | G2M checkpoint | 5% | 2.89E-09 |
| Hallmark | Mitotic spindle | 3% | 3.29E-04 |
| Hallmark | mTORC1 signaling | 3% | 3.29E-04 |
| Hallmark | Angiogenesis | 6% | 1.23E-02 |
| Hallmark | Complement | 2% | 2.91E-02 |
| Reactome | Neutrophil degranulation | 6% | 1.00E-31 |
| Reactome | Innate immune system | 2% | 9.07E-22 |
| Reactome | Antimicrobial peptides | 10% | 1.41E-12 |
| Reactome | Cell cycle | 2% | 4.74E-11 |
| Reactome | Homology directed repair | 4% | 8.26E-04 |

The genes present in the co-expressed modules shown in Figure 1 were subjected to overrepresentation analysis using the Molecular Signatures Database (MSigDB). Pathway datasets from hallmark gene sets and Reactome were used.

Gene ratio (k/K) refers to the overlap between the number of query genes (k) and the number of genes within each gene set from MSigDB (K).

FDR: This is the false discovery rate analog of hypergeometric p-value after correction for multiple hypothesis testing according to Benjamini and Hochberg (BH). We considered results with BH-adjusted p-values <0.05 as statistically significant. Each analysis is summarized with the top 5 pathways.

**Table S6**. **Gene module overrepresentation analysis of patients admitted to the ICU with sepsis stratified into clusters by percentage of MS1 cells using one-dimensional k-means clustering**

| **Module 1** | | | |
| --- | --- | --- | --- |
| **Gene Set Name** | **Pathway name** | **k/K** | **FDR** |
| Hallmark | Epithelial mesenchymal transition | 3% | 5.92E-05 |
| Hallmark | IL2/STAT5 signaling | 3% | 6.68E-04 |
| Hallmark | Adipogenesis | 2% | 4.18E-03 |
| Hallmark | Complement | 2% | 4.18E-03 |
| Hallmark | Kras signaling up | 2% | 4.18E-03 |
| Reactome | Hemostasis | 4% | 8.63E-24 |
| Reactome | Platelet activation signaling and aggregation | 6% | 4.72E-17 |
| Reactome | Response to elevated platelet cytosolic Ca2+ | 7% | 2.37E-09 |
| Reactome | Smooth muscle contraction | 14% | 9.38E-08 |
| Reactome | Formation of fibrin clot clotting cascade | 13% | 3.34E-06 |
| **Module 2** | | | |
| **Gene Set Name** | **Pathway name** | **k/K** | **FDR** |
| Hallmark | Allograft rejection | 7% | 7.91E-13 |
| Hallmark | IL2/STAT5 signaling | 4% | 1.18E-06 |
| Hallmark | Estrogen response early | 4% | 1.18E-06 |
| Hallmark | Myc targets v1 | 4% | 1.18E-06 |
| Hallmark | Interferon gamma response | 4% | 1.51E-05 |
| Reactome | Adaptive immune system | 2% | 2.66E-10 |
| Reactome | Cytokine signaling in immune system | 2% | 5.70E-07 |
| Reactome | Generation of second messenger molecules | 15% | 6.67E-07 |
| Reactome | TCR signaling | 6% | 9.50E-07 |
| Reactome | PD-1 signaling | 14% | 2.27E-04 |
| **Module 3** | | | |
| **Gene Set Name** | **Pathway name** | **k/K** | **FDR** |
| Hallmark | G2M checkpoint | 5% | 2.76E-09 |
| Hallmark | mTORC1 signaling | 3% | 3.21E-04 |
| Hallmark | Estrogen response late | 2% | 3.45E-03 |
| Hallmark | Angiogenesis | 6% | 1.21E-02 |
| Hallmark | Reactive oxygen species pathway | 4% | 1.98E-02 |
| Reactome | Neutrophil degranulation | 6% | 8.39E-32 |
| Reactome | Innate immune system | 2% | 8.27E-22 |
| Reactome | Antimicrobial peptides | 10% | 1.52E-12 |
| Reactome | Cell cycle | 2% | 4.38E-11 |
| Reactome | Cell cycle mitotic | 3% | 5.02E-10 |
| **Module 4** | | | |
| **Gene Set Name** | **Pathway name** | **k/K** | **FDR** |
| Hallmark | TNF signalling via NF-Κb | 5% | 1.74E-05 |
| Hallmark | Hypoxia | 4% | 9.49E-05 |
| Hallmark | Inflammatory response | 4% | 6.09E-04 |
| Hallmark | Adipogenesis | 3% | 3.56E-03 |
| Hallmark | Cholesterol homeostasis | 5% | 3.56E-03 |
| Reactome | Neutrophil degranulation | 4% | 1.27E-07 |
| Reactome | Innate immune system | 2% | 1.50E-06 |
| Reactome | Metabolism of lipids | 2% | 7.82E-04 |
| Reactome | Signaling by interleukins | 3% | 7.82E-04 |
| Reactome | Metabolic disorders of biological oxidation enzymes | 12% | 5.58E-03 |
| **Module 5** | | | |
| **Gene Set Name** | **Pathway name** | **k/K** | **FDR** |
| Hallmark | Complement | 7.50% | 8.48E-07 |
| Hallmark | IL2/STAT5 signaling | 7.04% | 2.82E-06 |
| Hallmark | mTORC1 signaling | 6.50% | 9.83E-06 |
| Hallmark | TNF signalling via NF-κB | 6.50% | 9.83E-06 |
| Hallmark | IL-6/JAK/STAT3 signaling | 6.90% | 2.59E-03 |
| Reactome | Cellular responses to stimuli | 5.21% | 3.80E-13 |
| Reactome | Post translational protein modification | 3.96% | 3.80E-13 |
| Reactome | Metabolism of amino acids and derivatives | 6.68% | 1.57E-09 |
| Reactome | Eukaryotic translation elongation | 14.89% | 1.57E-09 |
| Reactome | Innate immune system | 3.82% | 3.21E-09 |

The genes present in the co-expressed modules shown in Figure S5 were subjected to overrepresentation analysis using the Molecular Signatures Database (MSigDB). Pathway datasets from hallmark gene sets and Reactome were used.

Gene ratio (k/K) refers to the overlap between the number of query genes (k) and the number of genes within each gene set from MSigDB (K).

FDR: This is the false discovery rate analog of hypergeometric p-value after correction for multiple hypothesis testing according to Benjamini and Hochberg (BH). We considered results with BH-adjusted p-values <0.05 as statistically significant. Each analysis is summarized with the top 5 pathways.

**Table S7. Biomarker concentrations (median [IQR]) of patients admitted to the ICU with sepsis stratified into tertiles by percentage of MS1 cells**

| **Inflammatory response** | | | |
| --- | --- | --- | --- |
| **Biomarker** | **Low** (N = 111)^1^ | **Intermediate** (N = 111)^1^ | **High** (N = 110)^1^ |
| IL-8 (pg/mL) | 71.9 (30.0, 270.5) | 126.2 (49.6, 548.7) | 166.2 (55.3, 612.7) |
| IL-6 (pg/mL) | 137.5 (32.8, 447.3) | 186.7 (45.4, 1,013.8) | 204.7 (48.2, 1,224.0) |
| IL-10 (pg/mL) | 9.7 (3.6, 29.7) | 15.0 (4.5, 74.2) | 18.9 (6.0, 67.3) |
| NGAL (ng/mL) | 211.2 (120.7, 351.1) | 352.2 (188.3, 528.1) | 324.4 (177.7, 542.4) |
| MMP8 (ng/mL) | 2.3 (0.9, 5.2) | 4.3 (1.5, 15.5) | 6.5 (1.6, 16.9) |
| **Endothelial cell activation** | | | |
| sE-selectin (ng/mL) | 8.9 (4.4, 21.2) | 11.0 (3.6, 26.4) | 9.2 (4.6, 22.3) |
| sICAM-1 (ng/mL) | 134.6 (93.3, 248.1) | 179.1 (84.3, 280.5) | 175.4 (105.4, 317.4) |
| Fractalkine (pg/mL) | 23.4 (13.4, 40.0) | 24.1 (12.9, 55.5) | 30.9 (16.2, 57.6) |
| ANG2:ANG1 ratio | 2.1 (0.5, 8.0) | 4.0 (1.1, 18.1) | 4.3 (1.2, 16.8) |
| ANG1 (ng/mL) | 2.9 (1.0, 5.3) | 1.8 (0.7, 5.3) | 1.6 (0.6, 3.9) |
| ANG2 (ng/mL) | 5.7 (2.5, 13.3) | 9.0 (2.9, 19.1) | 7.5 (3, 15.9) |
| **Coagulation activation** | | | |
| Antitrombin (ng/mL) | 699.3 (532.7, 1106.5) | 629.2 (429.1, 963.3) | 571.4 (397.7, 975.7) |
| Protein C (ng/mL) | 124.5 (88.0, 160.4) | 107.0 (85.5, 144.2) | 97.0 (71.2, 130.6) |
| PT (sec) | 14.4 (12.2, 16.7) | 16.3 (13.5, 20.2) | 15.8 (13.3, 18.8) |
| Platelet count (10^9^/L) | 189.0 (127.8, 259.8) | 177.0 (110.0, 258.5) | 154.0 (94.0, 244.0) |
| D-dimer (µg/mL) | 7.4 (4, 17.2) | 11.8 (5.4, 21.7) | 11.11 (5, 19.6) |

Abbreviations: IL: interleukin; MMP8: matrix metalloproteinase 8; NGAL: neutrophil gelatinase-associated lipocalin; ANG1: angiopoietin 1; ANG2: angiopoietin 2; sE-selectin: soluble E-selectin; sICAM-1: soluble intercellular adhesion molecule 1; PT: prothrombin time.

**Table S8. Contribution of each biomarker to principal components 1 and 2 in each host response domain in patients admitted to the ICU with sepsis stratified into tertiles by percentage of MS1 cells**

| **Inflammatory response** | | |
| --- | --- | --- |
| **Biomarker** | **Contribution to PC1 (%)** | **Contribution to PC2 (%)** |
| IL-8 | 22.40 | 12.54 |
| IL-6 | 22.11 | 14.60 |
| IL-10 | 20.85 | 16.86 |
| NGAL | 18.89 | 23.36 |
| MMP8 | 15.75 | 32.64 |
| **Endothelial cell activation** | | |
| **Biomarker** | **Contribution to PC1 (%)** | **Contribution to PC2 (%)** |
| sE-selectin | 25.82 | 15.35 |
| sICAM-1 | 25.03 | 17.62 |
| Fractalkine | 16.06 | 8.44 |
| ANG2:ANG1 | 13.06 | 26.62 |
| ANG1 | 11.33 | 28.98 |
| ANG2 | 8.71 | 2.98 |
| **Coagulation activation** | | |
| **Biomarker** | **Contribution to PC1 (%)** | **Contribution to PC2 (%)** |
| Antitrombin | 26.78 | 22.94 |
| Protein C | 26.46 | 0.97 |
| PT | 24.27 | 8.18 |
| Platelet count | 19.42 | 11.13 |
| D-Dimer | 3.06 | 56.78 |

Abbreviations: IL: Interleukin; MPP8: matrix metallopeptidase 8; NGAL: Neutrophil gelatinase-associated lipocalin; ANG1: angiopoietin 1; ANG2: angiopoietin 2; sE-selectin: soluble E-selectin; sICAM-1: soluble intercellular adhesion molecule 1; PT: prothrombin time.

**Table S9. Baseline characteristics and outcomes of patients admitted to the ICU with sepsis and critically ill patients without infection**

| **Characteristic** | **Sepsis** (N = 332)*^1^* | **Non-Inf ICU*** (**N = 215)*^1^* | **p-value** |
| --- | --- | --- | --- |
| MS1 relative percentage | 23.7 (20.2, 27.7) | 24.0 (20.5, 27.6) | 0.99 |
| **Demographics** | | | |
| Age years | 64.0 (55.0, 73.3) | 64.0 (51.5, 73.0) | 0.4 |
| White race | 282 (85%) | 185 (86%) | 0.8 |
| Male sex | 197 (59%) | 134 (62%) | 0.5 |
| Admission type, surgery | 91 (27%) | 107 (50%) | **<0.00001** |
| BMI | 24.8 (22.5, 28.4) | 25.8 (23.0, 29.3) | 0.15 |
| **Comorbidity** | | | |
| Charlson score | 4.0 (2.0, 5.0) | 4.0 (2.0, 5.0) | 0.5 |
| Cardiovascular insufficiency | 16 (4.8%) | 16 (7.4%) | 0.3 |
| Respiratory insufficiency | 24 (7.2%) | 11 (5.1%) | 0.4 |
| Renal insufficiency | 38 (11%) | 14 (6.5%) | 0.08 |
| Hypertension | 89 (27%) | 81 (38%) | **0.01** |
| Diabetes mellitus | 59 (18%) | 48 (22%) | 0.2 |
| COPD | 49 (15%) | 16 (7.4%) | **0.01** |
| Cerebrovascular disease | 27 (8.1%) | 12 (5.6%) | 0.3 |
| **Disease severity on admission** | | | |
| SOFA Score | 7.0 (5.0, 9.0) | 6.0 (4.0, 9.0) | 0.06 |
| APACHE IV Score | 79.0 (62.0, 98.5) | 72.0 (51.0, 100.5) | 0.08 |
| APS | 67.0 (50.0, 85.3) | 62.0 (43.0, 89.5) | 0.1 |
| ARDS | 90 (27%) | 14 (6.5%) | **<0.00001** |
| AKI | 127 (38%) | 71 (34%) | 0.28 |
| Shock | 124 (37%) | 80 (37%) | >0.9 |
| **Outcomes** | | | |
| Hospital LOS, days | 16.0 (8.0, 38.0) | 14.0 (6.0, 32.0) | **0.04** |
| ICU LOS, days | 5.0 (2.0, 11.0) | 4.0 (2.0, 9.0) | 0.09 |
| **ICU‑acquired complications** | | | |
| ARDS | 41 (12%) | 17 (7.9%) | 0.1 |
| Infection | 39 (12%) | 43 (20%) | **0.01** |
| **Mortality** | | | |
| Death in ICU | 66 (20%) | 49 (23%) | 0.5 |
| 30‐day mortality | 93 (28%) | 56 (26%) | 0.68 |
| 60‐day mortality | 109 (33%) | 72 (33%) | >0.9 |
| 90‐day mortality | 117 (35%) | 74 (34%) | >0.9 |

^1^ Median (IQR); n / N (%)

* Non-Inf ICU: critically ill patients without infection

Abbreviations: AKI: acute kidney injury, APACHE: acute physiology and chronic health evaluation, APS: acute physiology scores, ARDS: acute respiratory distress syndrome, BMI: body mass index, COPD: chronic obstructive pulmonary disease, LOS: length of stay, SOFA: sequential organ failure assessment

**Table S10. Baseline characteristics and outcomes of non-infected critically ill patients stratified into tertiles by percentage of MS1 cells**

| **Characteristic** | **Low** (N = 72)*^1^* | **Intermediate** (N = 72)*^1^* | **High (**N = 71)*^1^* | **p-value** |
| --- | --- | --- | --- | --- |
| Percentage of MS1 cells | 19.1 (17.2, 20.5)^a^ | 24.0 (22.8, 24.9)^b^ | 30.0 (27.6, 33.0)^c^ | **<0.00001** |
| **Demographics** | | | | |
| Age years | 63.0 (48.0, 71.0) | 65.5 (53.5, 73.3) | 63.0 (53.5, 73.5) | 0.4 |
| White race | 60 (83%) | 63 (88%) | 62 (87%) | 0.7 |
| Male sex | 38 (53%) | 48 (67%) | 48 (68%) | 0.1 |
| Admission type, surgery | 31 (43%) | 41 (57%) | 35 (49%) | 0.2 |
| BMI | 26.4 (23.2, 30.4) | 25.1 (22.5, 28.1) | 26.1 (23.1, 28.8) | 0.5 |
| **Comorbidity** | | | | |
| Charlson score | 3.5 (2.0, 5.0) | 4.0 (2.8, 5.3) | 4.0 (2.0, 5.5) | 0.2 |
| Cardiovascular insufficiency | 6 (8.3%) | 6 (8.3%) | 4 (5.6%) | 0.8 |
| Respiratory insufficiency | 3 (4.2%) | 2 (2.8%) | 6 (8.5%) | 0.3 |
| Renal insufficiency | 7 (9.7%) | 2 (2.8%) | 5 (7.0%) | 0.2 |
| Hypertension | 27 (38%) | 32 (44%) | 22 (31%) | 0.3 |
| Diabetes mellitus | 16 (22%) | 22 (31%) | 10 (14%) | 0.06 |
| COPD | 2 (2.8%)^a^ | 4 (5.6%)^a,b^ | 10 (14%)^b^ | **0.02** |
| Cerebrovascular disease | 4 (5.6%) | 4 (5.6%) | 4 (5.6%) | >0.9 |
| **Disease severity on admission** | | | | |
| SOFA Score | 6.0 (3.0, 8.0)^a^ | 7.0 (5.0, 9.0)^a,b^ | 7.0 (5.0, 10.0)^b^ | **0.01** |
| APACHE IV Score | 66.0 (47.8, 90.8) | 73.0 (52.0, 101.3) | 84.0 (56.0, 112.5) | 0.08 |
| APS | 54.0 (39.5, 76.8) | 62.0 (43.0, 89.0) | 74.0 (49.0, 99.5) | 0.08 |
| ARDS | 3 (4.2%) | 5 (6.9%) | 6 (8.5%) | 0.6 |
| AKI | 24 (34%) | 26 (37%) | 21 (29%) | 0.8 |
| Shock | 25 (35%) | 24 (33%) | 31 (44%) | 0.4 |
| **Outcomes** | | | | |
| Hospital LOS, days | 12.5 (6.0, 27.5) | 16.5 (6.8, 32.5) | 12.0 (5.5, 32.0) | 0.4 |
| ICU LOS, days | 3.0 (2.0, 5.0) | 4.0 (2.0, 10.0) | 5.0 (3.0, 11.5) | 0.1 |
| **ICU‑acquired complications** | | | | |
| ARDS | 4 (5.6%) | 7 (9.7%) | 6 (8.5%) | 0.6 |
| Infection | 11 (15%) | 17 (24%) | 15 (21%) | 0.4 |
| **Mortality** | | | | |
| Death in ICU | 12 (17%) | 19 (26%) | 18 (25%) | 0.3 |
| 30‐day mortality | 13 (18%) | 21 (29%) | 22 (31%) | 0.16 |
| 60‐day mortality | 18 (25%)^a^ | 23 (32%)^a,b^ | 31 (44%)^b^ | **0.05** |
| 90‐day mortality | 19 (26%)^a^ | 23 (32%)^a,b^ | 32 (45%)^b^ | **0.05** |

^1^ Median (IQR); n / N (%)

*^a-c^* Groups that have no superscript in common are significantly different from each other after post-hoc tests with Benjamini-Hochberg correction (p < 0.05).

Abbreviations: AKI = acute kidney injury, APACHE = acute physiology and chronic health evaluation, APS = acute physiology scores, ARDS = acute respiratory distress syndrome, BMI = body mass index, COPD = chronic obstructive pulmonary disease, LOS = length of stay, SOFA = sequential organ failure assessment

**Table S11. Admission diagnoses of non-infected critically ill patients stratified into tertiles by percentage of MS1 cells**

| **Diagnosis** | **Low** (N = 72) | **Intermediate** (N = 72) | **High** (N = 71) | **p-value** |
| --- | --- | --- | --- | --- |
| Cardiac arrest | 13 (18%) | 13 (18%) | 15 (21%) | 0.86 |
| Cardiac failure | 8 (11%) | 1 (1.4%) | 8 (11%) | 0.06 |
| Cardiovascular surgery | 12 (17%) | 16 (22%) | 15 (21%) | 0.67 |
| Cerebrovascular disease | 7 (9.7%) | 3 (4.2%) | 5 (7.0%) | 0.42 |
| Gastrointestinal surgery | 14 (19%) | 17 (24%) | 8 (11%) | 0.15 |
| Respiratory failure | 5 (6.9%) | 6 (8.3%) | 4 (5.6%) | 0.81 |
| Trauma | 3 (4.2%) | 9 (13%) | 11 (15%) | 0.07 |
| Other^1^ | 10 (14%) | 7 (9.7%) | 5 (7.0%) | 0.39 |

^1^ Other: 20 different admission diagnoses (of which 18 once and 2 twice)

**Table S12. Optimal number of clusters based on the percentage of MS1 cells in non-infected critically ill patients: consensus-based algorithm results**

| Number of Clusters | Method | Package | Duration |
| --- | --- | --- | --- |
| 1 | GapMaechler2012 | easystats | 0.47 |
| 1 | GapDudoit2002 | easystats | 0.46 |
| 1 | Frey | NbClust | 0.05 |
| 1 | Mixture (E) | mclust | 0.13 |
| 1 | Mixture (V) | mclust | 0.13 |
| 2 | Elbow | easystats | 0.10 |
| 2 | Silhouette | easystats | 0.02 |
| 2 | kl | NbClust | 0.04 |
| 2 | CCC | NbClust | 0.02 |
| 2 | Duda | NbClust | 0.01 |
| 2 | Pseudot2 | NbClust | 0.01 |
| 2 | Beale | NbClust | 0.01 |
| 2 | Mcclain | NbClust | 0.15 |
| 3 | Hartigan | NbClust | 0.03 |
| 3 | Scott | NbClust | 0.01 |
| 3 | Tracew | NbClust | 0.02 |
| 3 | Ball | NbClust | 0.01 |
| 3 | PtBiserial | NbClust | 0.38 |
| 4 | SDindex | NbClust | 0.01 |
| 5 | Ratkowsky | NbClust | 0.01 |
| 8 | Cindex | NbClust | 0.01 |
| 9 | Ch | NbClust | 0.03 |
| 9 | Marriot | NbClust | 0.01 |
| 9 | Friedman | NbClust | 0.01 |
| 9 | Rubin | NbClust | 0.01 |
| 9 | DB | NbClust | 0.01 |
| 9 | Dunn | NbClust | 0.01 |
| 9 | SDbw | NbClust | 0.08 |

**Table S13. Baseline characteristics and outcomes of non-infected critically ill patients stratified into clusters by percentage of MS1 cells using one-dimensional k-means clustering**

| **Characteristic** | **Cluster 1** (Low, N = 103)*^1^* | **Cluster 2** (High, N = 112)*^1^* | **p-value** |
| --- | --- | --- | --- |
| MS1 relative percentage | 20.4 (18.0, 22.0) | 27.4 (25.4, 31.0) | **<0.00001** |
| **Demographics** | | | |
| Age years | 61.0 (48.0, 71.0) | 67.0 (54.8, 75.0) | **0.01** |
| White race | 88 (85%) | 97 (87%) | 0.95 |
| Male sex | 61 (59%) | 73 (65%) | 0.44 |
| Admission type, surgery | 49 (48%) | 58 (52%) | 0.63 |
| BMI | 25.1 (22.9, 29.9) | 26.1 (23.1, 28.4) | 0.77 |
| **Comorbidity** | | | |
| Charlson score | 3.0 (2.0, 5.0) | 4.0 (3.0, 6.0) | **0.02** |
| Cardiovascular insufficiency | 9 (8.7%) | 7 (6.3%) | 0.66 |
| Respiratory insufficiency | 4 (3.9%) | 7 (6.3%) | 0.63 |
| Renal insufficiency | 8 (7.8%) | 6 (5.4%) | 0.66 |
| Hypertension | 41 (40%) | 40 (36%) | 0.63 |
| Diabetes mellitus | 27 (26%) | 21 (19%) | 0.25 |
| COPD | 4 (3.9%) | 12 (11%) | 0.10 |
| Cerebrovascular disease | 7 (6.8%) | 5 (4.5%) | 0.6 |
| **Disease severity on admission** | | | |
| SOFA Score | 6.0 (3.0, 8.0) | 7.0 (5.0, 10.0) | **0.003** |
| APACHE IV Score | 67.0 (46.5, 90.5) | 82.5 (55.8, 107.0) | **0.004** |
| APS | 54.0 (40.0, 81.0) | 70.5 (49.0, 96.5) | **0.01** |
| ARDS | 5 (4.9%) | 9 (8.0%) | 0.5 |
| AKI | 32 (31%) | 39 (34%) | 0.52 |
| Shock | 32 (31%) | 48 (43%) | 0.09 |
| **Outcomes** | | | |
| Hospital LOS, days | 12.0 (6.0, 26.5) | 15.0 (5.8, 36.8) | 0.36 |
| ICU LOS, days | 4.0 (2.0, 6.5) | 5.0 (2.0, 11.0) | 0.08 |
| **ICU‑acquired complications** | | | |
| ARDS | 8 (7.8%) | 9 (8.0%) | >0.99 |
| ICU‑acquired infection | 17 (17%) | 26 (23%) | 0.29 |
| **Mortality** | | | |
| Death in ICU | 19 (18%) | 30 (27%) | 0.19 |
| 30‐day mortality | 22 (21%) | 34 (30%) | 0.1 |
| 60‐day mortality | 28 (27%) | 44 (39%) | 0.08 |
| 90‐day mortality | 29 (28%) | 45 (40%) | 0.08 |

^1^ Median (IQR); n / N (%)

Abbreviations: AKI: acute kidney injury, APACHE: acute physiology and chronic health evaluation, APS: acute physiology scores, ARDS: acute respiratory distress syndrome, BMI: body mass index, COPD: chronic obstructive pulmonary disease, LOS: length of stay, SOFA: sequential organ failure assessment

**Table S14**. **Gene module overrepresentation analysis of non-infected critically ill patients stratified into tertiles by percentage of MS1 cells**

| **Module 1** | | | |
| --- | --- | --- | --- |
| **Gene Set Name** | **Pathway names** | **k/K** | **FDR** |
| Hallmark | Allograft rejection | 8% | 6.76E-11 |
| Hallmark | Estrogen response early | 7% | 6.47E-09 |
| Hallmark | IL2/STAT5 signaling | 5% | 5.78E-06 |
| Hallmark | Interferon gamma response | 5% | 3.34E-05 |
| Hallmark | MYC targets v1 | 5% | 3.34E-05 |
| Reactome | Adaptive immune system | 3% | 2.48E-10 |
| Reactome | Cytokine Signaling in Immune system | 3% | 9.04E-08 |
| Reactome | RNA polymerase II transcription | 2% | 7.97E-04 |
| Reactome | TCR signaling | 6% | 1.95E-03 |
| Reactome | MHC class II antigen presentation | 6% | 1.95E-03 |
| **Module 2** | | | |
| **Gene Set Name** | **Pathway names** | **k/K** | **FDR** |
| Hallmark | MYC targets v1 | 5% | 5.86E-07 |
| Hallmark | mTORC1 signaling | 3% | 1.85E-03 |
| Hallmark | Fatty acid metabolism | 3% | 3.60E-03 |
| Hallmark | Ultraviolet response UP | 3% | 3.60E-03 |
| Hallmark | Elongation factor 2 signaling | 3% | 9.05E-03 |
| Reactome | Cellular response to heat stress | 9% | 6.56E-07 |
| Reactome | Regulation of HSF1-mediated heat shock response | 9% | 4.15E-05 |
| Reactome | Attenuation phase | 18% | 5.45E-05 |
| Reactome | Cellular responses to stimuli | 2% | 9.69E-05 |
| Reactome | RNA polymerase II transcription | 1% | 1.18E-03 |
| **Module 3** | | | |
| **Gene Set Name** | **Pathway names** | **k/K** | **FDR** |
| Hallmark | Interferon-gamma response | 18% | 4.52E-68 |
| Hallmark | Interferon-alpha response | 30% | 4.40E-63 |
| Hallmark | Allograft rejection | 2% | 1.44E-02 |
| Hallmark | Complement | 2% | 1.44E-02 |
| Hallmark | KRAS signaling DN | 2% | 1.44E-02 |
| Reactome | Interferon signaling | 11% | 1.18E-36 |
| Reactome | Interferon alpha/beta signaling | 22% | 1.14E-29 |
| Reactome | Antiviral mechanism by IFN-stimulated genes | 12% | 3.05E-15 |
| Reactome | Interferon-gamma signaling | 11% | 1.02E-14 |
| Reactome | OAS antiviral response | 56% | 8.60E-11 |
| **Module 4** | | | |
| **Gene Set Name** | **Pathway names** | **k/K** | **FDR** |
| Hallmark | MYC targets v1 | 5% | 1.43E-08 |
| Hallmark | Oxidative phosphorylation | 4% | 4.16E-06 |
| Hallmark | p53 pathway | 2% | 1.19E-02 |
| Hallmark | DNA repair | 2% | 4.31E-02 |
| Reactome | Eukaryotic translation elongation | 19% | 5.04E-28 |
| Reactome | SRP-dependent cotranslational protein targeting to membrane | 16% | 9.17E-27 |
| Reactome | Selenoamino acid metabolism | 15% | 1.41E-26 |
| Reactome | Response of EIF2AK4 (GCN2) to amino acid deficiency | 17% | 8.26E-26 |
| Reactome | Translation | 7% | 6.05E-23 |
| **Module 5** | | | |
| **Gene Set Name** | **Pathway names** | **k/K** | **FDR** |
| Hallmark | TNF signalling via NF-κB | 9% | 5.72E-16 |
| Hallmark | Inflammatory response | 8% | 2.36E-12 |
| Hallmark | Hypoxia | 5% | 1.23E-05 |
| Hallmark | Cholesterol homeostasis | 8% | 2.31E-05 |
| Hallmark | Epithelial mesenchymal transition | 4% | 4.09E-05 |
| Reactome | Neutrophil degranulation | 6% | 3.99E-22 |
| Reactome | Innate immune system | 3% | 5.20E-18 |
| Reactome | Interleukin-4 and Interleukin-13 signaling | 7% | 4.72E-05 |
| Reactome | Signaling by interleukins | 3% | 2.84E-04 |
| Reactome | Cytokine Signaling in Immune system | 2% | 2.84E-04 |

The genes present in the co-expressed modules shown in Figure 5 were subjected to overrepresentation analysis using the Molecular Signatures Database (MSigDB). Pathway datasets from hallmark gene sets and Reactome were used.

Gene ratio (k/K) refers to the overlap between the number of query genes (k) and the number of genes within each gene set from MSigDB (K).

FDR: This is the false discovery rate analog of hypergeometric p-value after correction for multiple hypothesis testing according to Benjamini and Hochberg (BH). We considered results with BH-adjusted p-values <0.05 as statistically significant. Each analysis is summarized with the top 5 pathways.

**Table S15**. **Gene module overrepresentation analysis of non-infected critically ill patients stratified into clusters by percentage of MS1 cells using one-dimensional k-means clustering**

| **Module 1** | | | |
| --- | --- | --- | --- |
| **Gene Set Name** | **Pathway names** | **k/K** | **FDR** |
| Hallmark | Oxidative phosphorylation | 4% | 4.02E-06 |
| Hallmark | p53 pathway | 2% | 1.17E-02 |
| Hallmark | DNA repair | 2% | 4.24E-02 |
| Reactome | Eukaryotic translation elongation | 19% | 4.69E-28 |
| Reactome | SRP-dependent cotranslational protein targeting to membrane | 16% | 8.55E-27 |
| Reactome | Selenoamino acid metabolism | 15% | 1.31E-26 |
| Reactome | Eukaryotic translation initiation | 14% | 9.93E-25 |
| Reactome | Translation | 7% | 5.59E-23 |
| **Module 2** | | | |
| **Gene Set Name** | **Pathway names** | **k/K** | **FDR** |
| Hallmark | MYC targets v1 | 5% | 5.58E-07 |
| Hallmark | TNF signalling via NF-Κb | 4% | 4.57E-05 |
| Hallmark | IL2/STAT5 signaling | 3% | 1.80E-03 |
| Hallmark | Interferon-alpha response | 3% | 3.70E-02 |
| Hallmark | mTORC1 signaling | 3% | 1.80E-03 |
| Reactome | Cellular response to heat stress | 9% | 6.42E-07 |
| Reactome | Regulation of HSF1-mediated heat shock response | 9% | 4.10E-05 |
| Reactome | Attenuation phase | 18% | 5.43E-05 |
| Reactome | HSF1 activation | 16% | 6.98E-05 |
| Reactome | Metabolism of RNA | 2% | 7.80E-03 |
| **Module 3** | | | |
| **Gene Set Name** | **Pathway names** | **k/K** | **FDR** |
| Hallmark | Allograft rejection | 8% | 6.28E-11 |
| Hallmark | IL2/STAT5 signaling | 5% | 5.51E-06 |
| Hallmark | Interferon-gamma response | 5% | 3.21E-05 |
| Hallmark | MYC targets v1 | 5% | 3.21E-05 |
| Hallmark | Elongation factor 2 signaling | 4% | 1.90E-04 |
| Reactome | Adaptive immune system | 3% | 2.24E-10 |
| Reactome | Binding of TCF/LEF:CTNNB1 to target gene promoters | 50% | 3.38E-05 |
| Reactome | RUNX3 regulates WNT signaling | 50% | 3.38E-05 |
| Reactome | Generation of second messenger molecules | 13% | 7.95E-04 |
| Reactome | MHC class II antigen presentation | 6% | 1.95E-03 |
| **Module 4** | | | |
| **Gene Set Name** | **Pathway names** | **k/K** | **FDR** |
| Hallmark | Complement | 9% | 5.45E-10 |
| Hallmark | TNF signalling via NF-Κb | 7% | 1.84E-06 |
| Hallmark | mTORC1 signaling | 6% | 9.21E-06 |
| Hallmark | Androgen response | 8% | 7.13E-05 |
| Hallmark | IL-6/JAK/STAT3 signaling | 8% | 1.99E-04 |
| Reactome | Toll Like Receptor 9 (TLR9) Cascade | 13% | 3.07E-09 |
| Reactome | Innate immune system | 3% | 3.19E-09 |
| Reactome | Toll Like Receptor TLR1:TLR2 Cascade | 12% | 3.19E-09 |
| Reactome | MyD88-independent TLR4 cascade | 12% | 1.44E-08 |
| Reactome | Toll like receptor cascades | 9% | 1.82E-08 |
| **Module 5** | | | |
| **Gene Set Name** | **Pathway names** | **k/K** | **FDR** |
| Hallmark | TNF signalling via NF-Κb | 9% | 5.23E-16 |
| Hallmark | Inflammatory response | 8% | 2.19E-12 |
| Hallmark | Hypoxia | 5% | 1.18E-05 |
| Hallmark | Cholesterol homeostasis | 8% | 2.24E-05 |
| Hallmark | IL2/STAT5 signaling | 4% | 3.93E-05 |
| Reactome | Neutrophil degranulation | 6% | 3.29E-22 |
| Reactome | Innate immune system | 3% | 4.60E-18 |
| Reactome | Interleukin-4 and Interleukin-13 signaling | 7% | 4.64E-05 |
| Reactome | Cytokine signaling in immune system | 2% | 7.25E-04 |
| Reactome | Antimicrobial peptides | 6% | 2.76E-03 |

The genes present in the co-expressed modules shown in Figure S9 were subjected to overrepresentation analysis using the Molecular Signatures Database (MSigDB). Pathway datasets from hallmark gene sets and Reactome were used.

Gene ratio (k/K) refers to the overlap between the number of query genes (k) and the number of genes within each gene set from MSigDB (K).

FDR: This is the false discovery rate analog of hypergeometric p-value after correction for multiple hypothesis testing according to Benjamini and Hochberg (BH). We considered results with BH-adjusted p-values <0.05 as statistically significant. Each analysis is summarized with the top 5 pathways.

**Supplementary Figures**


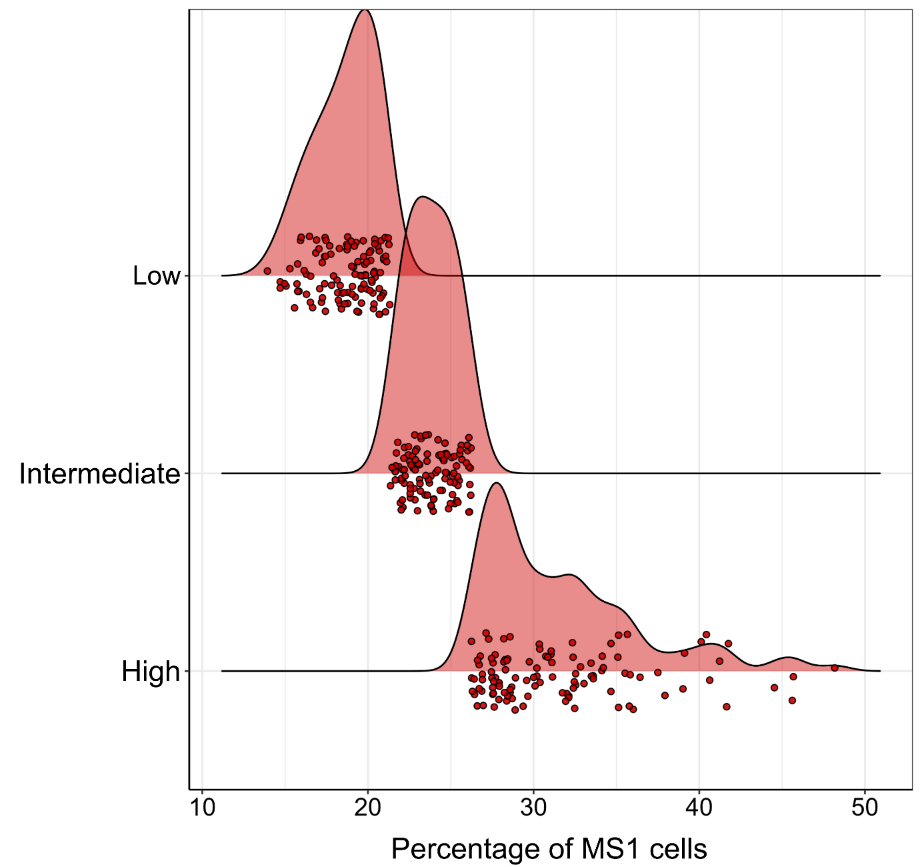


**Figure S1. Density Distribution plot.**

**Description Figure S1.** Distribution of MS1 percentages in the sepsis cohort stratified into tertiles by percentage of MS1 cells


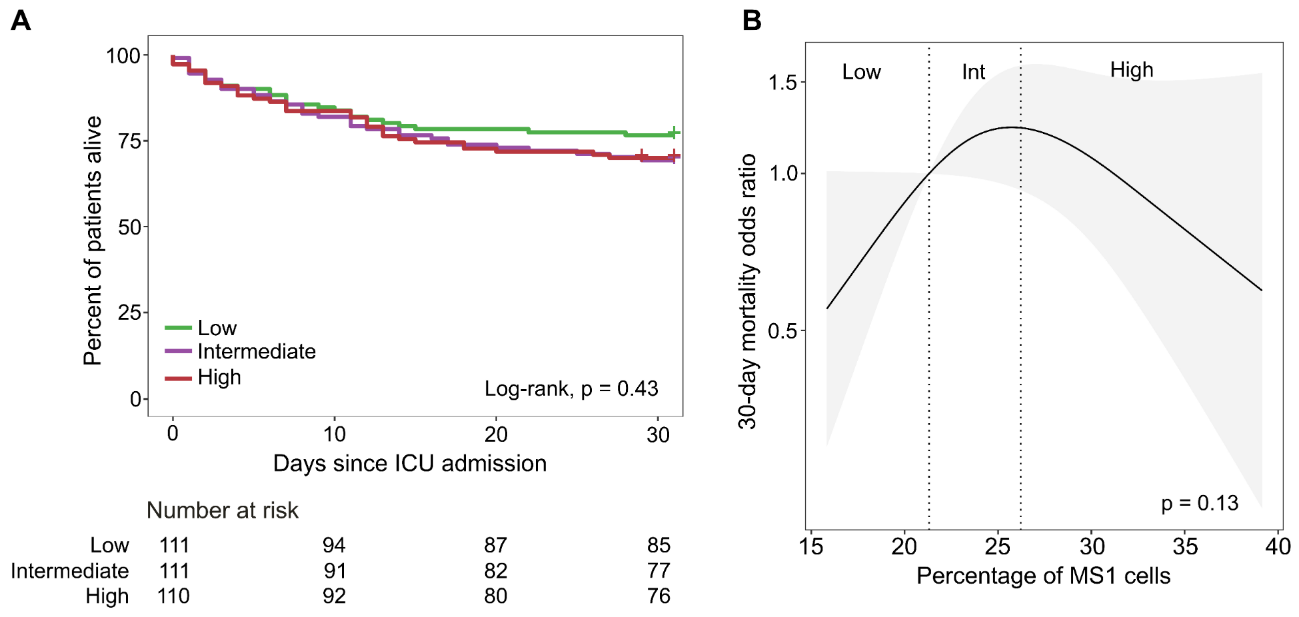


**Figure S2. Mortality analysis of patients with sepsis stratified into tertiles by percentage of MS1 cells**

**Description Figure S2: A)** Probability of 30-day survival. Thirty-day Kaplan–Meier survival curve of critically ill sepsis patients stratified by MS1 groups. **B)** The risk of 30-day mortality was modeled with the percentage of MS1 cells as a continuous variable. Given the non-linear relationship between MS1 cell percentage and mortality, a restricted cubic spline function with three inner knots at default quantile locations was used. To calculate the odds ratio, the reference was set to 21.31, which represents the maximum value in the low MS1 group. The gray shading represents the 95% confidence interval of the 30-day mortality odds ratio.


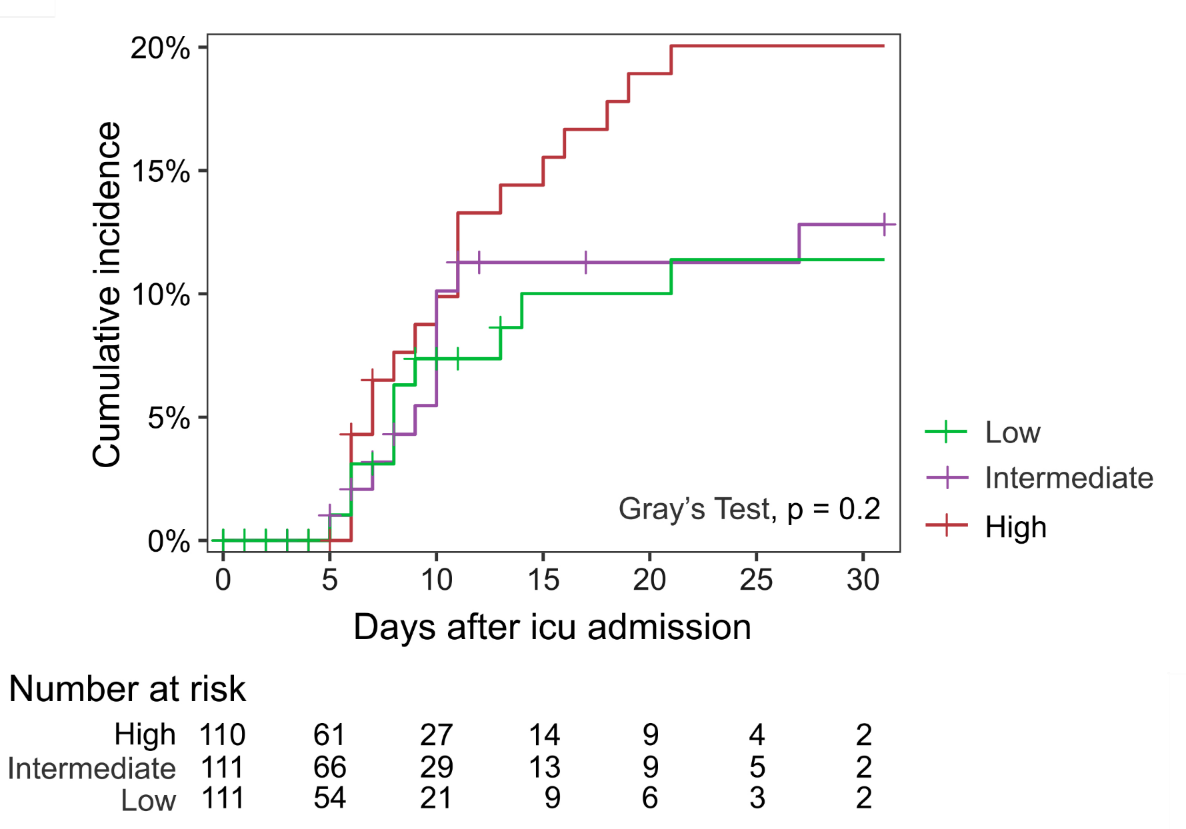


**Figure S3. Cumulative incidence of ICU-acquired infections in patients with sepsis stratified into tertiles by percentage of MS1 cells**

**Description Figure S3:** The graph illustrates the cumulative incidence (in days) of ICU-acquired infections; the p-value was computed using Gray's competing risks analysis.


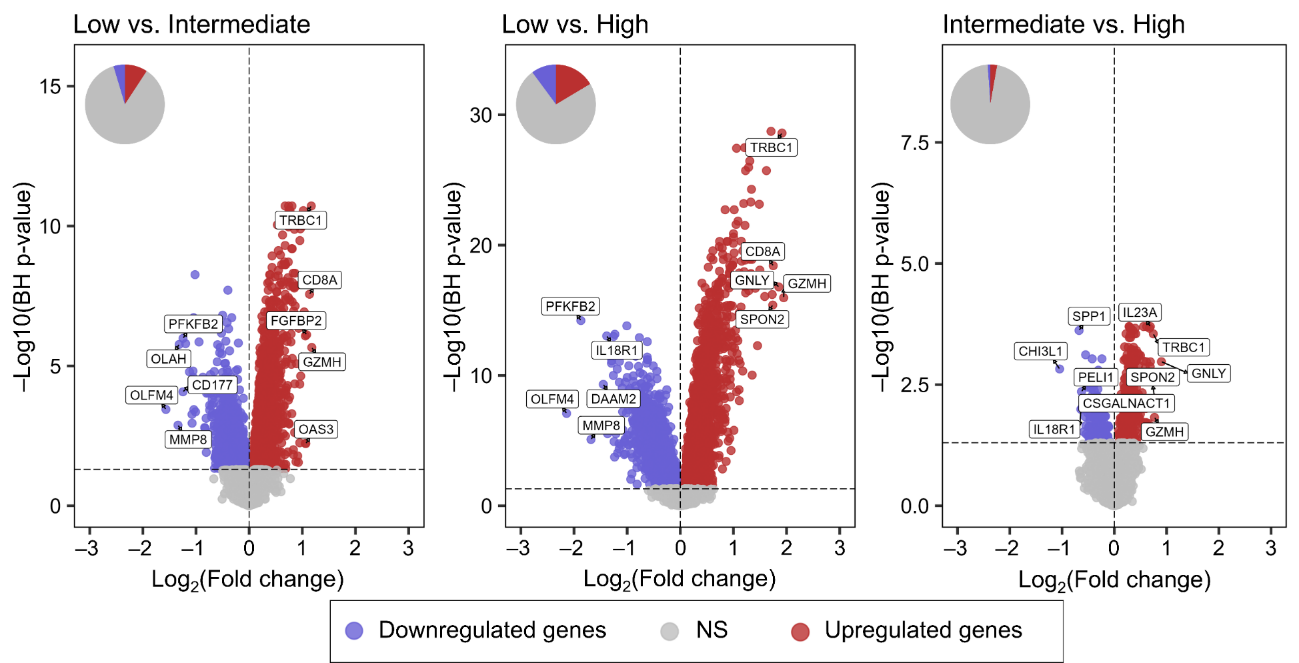


**Figure S4. Volcano plots of differential gene expression of patients admitted to the ICU with sepsis stratified into tertiles by percentage of MS1 cells**

**Description Figure S4:** Volcano plots illustrating the differences in leukocyte genomic responses among patients with varying MS1 cell levels. Genes are identified as differentially expressed with a BH adjusted p-value of less than 0.05. Gene names indicate the top five upregulated (in red) and top five downregulated (in blue) genes. Gray dots represent genes that were not differentially expressed between groups. Pie charts show the extent of gene expression differences between indicated MS1 groups.


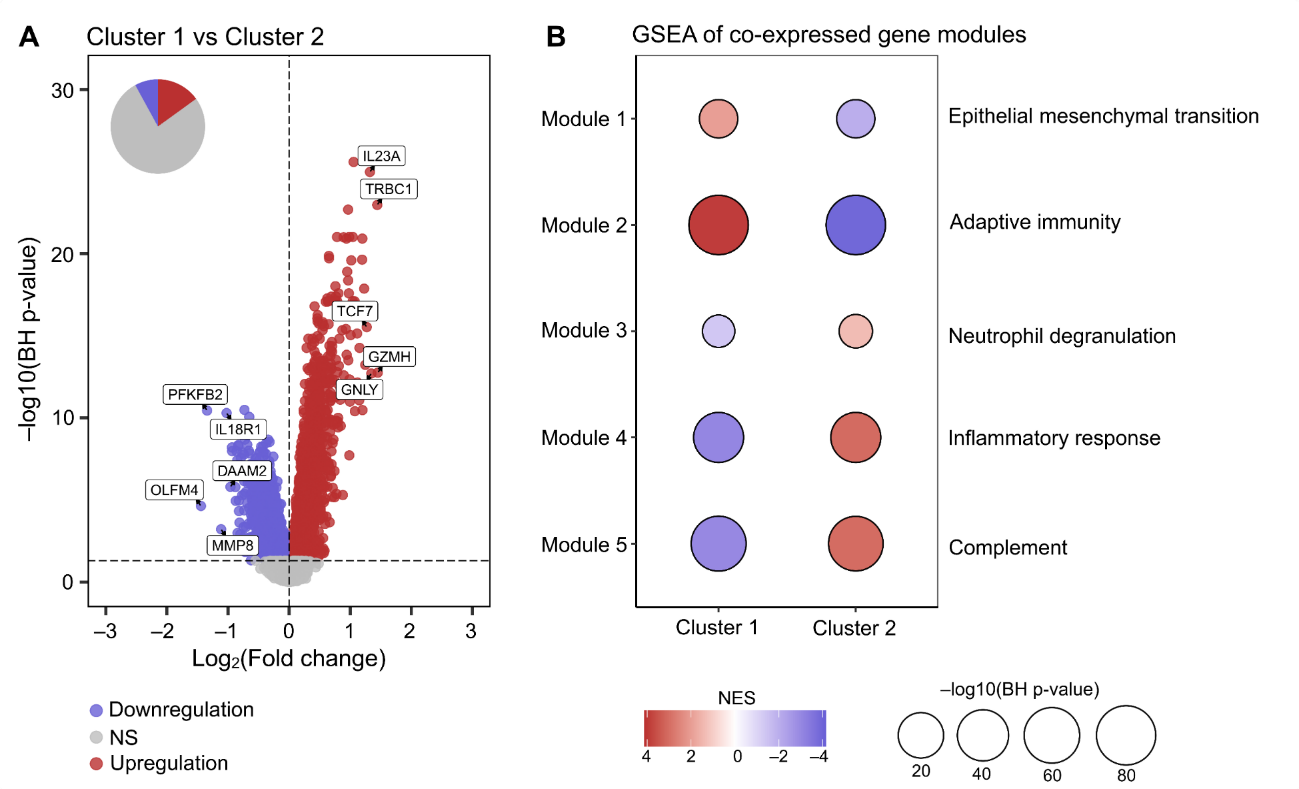


**Figure S5**. **Gene expression differences of patients admitted to the ICU with sepsis stratified into clusters by percentage of MS1 cells using one-dimensional k-means clustering**

**Description Figure S5: A)** Volcano plots illustrating the differences in leukocyte genomic responses among patients classified as cluster 1 (low MS1) and cluster 2 (high MS1). Genes are identified as differentially expressed with a BH adjusted p-value of less than 0.05. Gene names indicate the top five upregulated (in red) and top five downregulated (in blue) genes. Gray dots represent genes that were not differentially expressed between groups. Pie chart shows the extent of gene expression differences. **B)** The co-expression module identification analysis revealed distinct gene modules based on MS1 cell percentages in patients with sepsis. Patients were categorized as cluster 1 (low MS1) and cluster 2 (high MS1). The size of each circle in the graph is proportional to -log10(BH adjusted p-value), and the color represents the normalized enrichment score (NES). Blue indicates a decreased NES, and red represents an increased NES.


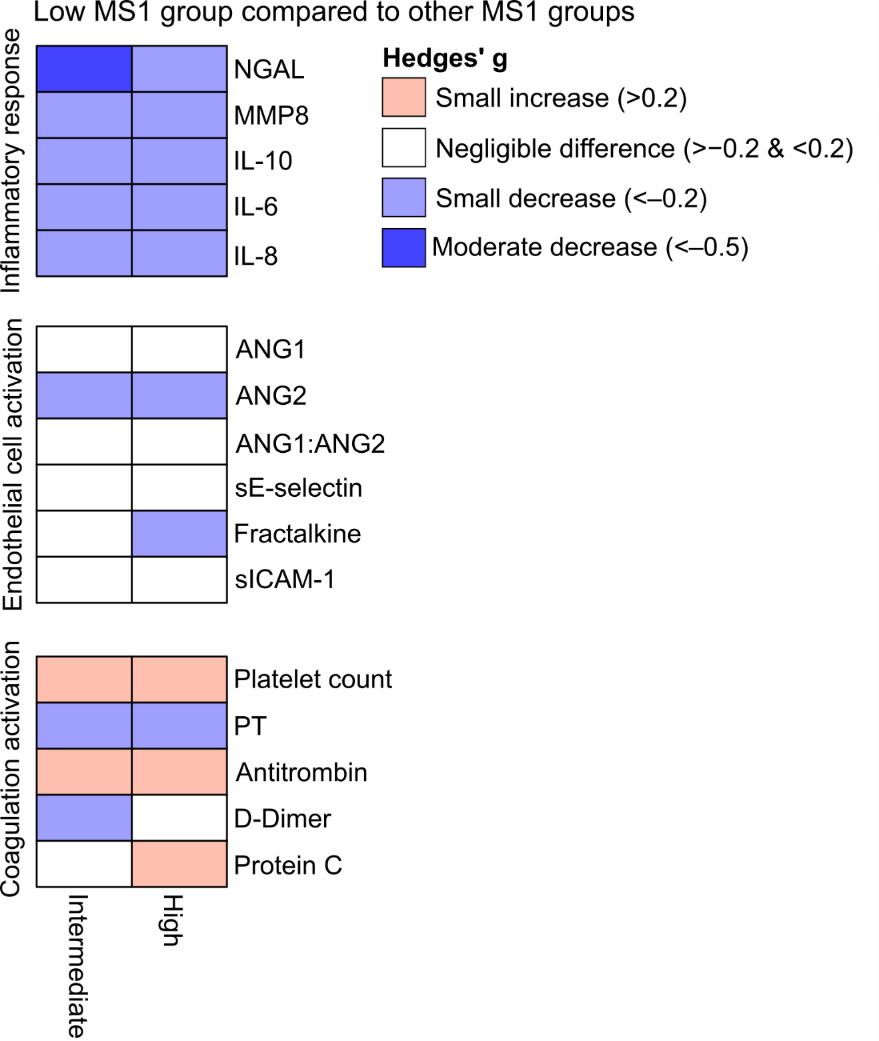


**Figure S6**. **Association of host response biomarkers of patients admitted to the ICU with sepsis stratified into tertiles by percentage of MS1 cells**

**Description Figure S6:** Heatmap with a visual representation of the magnitude of differences in biomarkers (quantified using Hedges’ g) between sepsis patients in the low MS1 group as compared with the intermediate and high MS1 groups. Red signifies increased biomarker levels in the low MS1 group, blue indicates decreased levels in the low MS1 group.


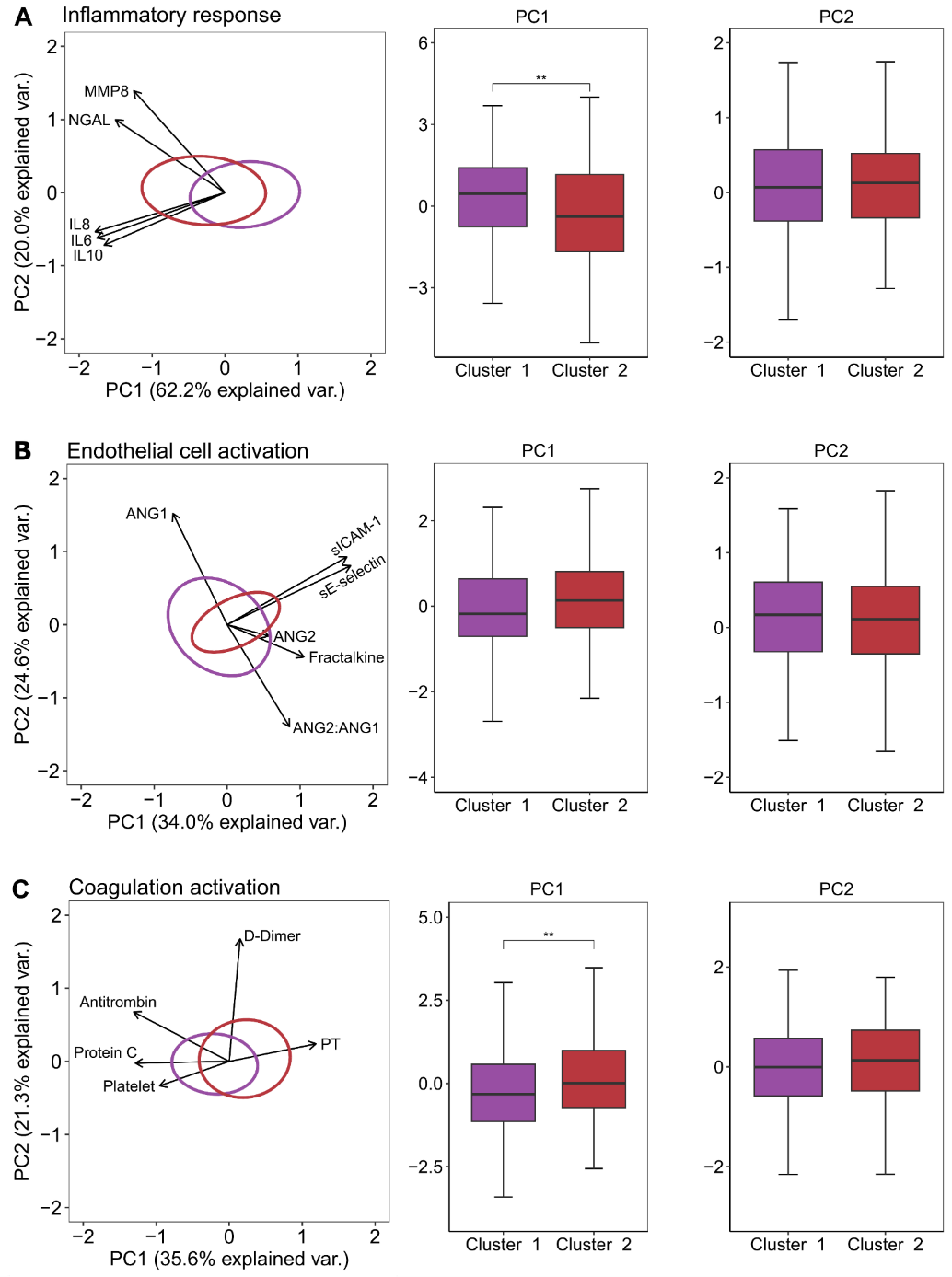


**Figure S7. Distinctive and overlapping host response biomarker profiles in plasma in patients admitted to the ICU with sepsis stratified into clusters by percentage of MS1 cells using one-dimensional k-means clustering**

**Description Figure S7:** Principal Component Analysis (PCA) in which principal components (PC) 1 and 2 are plotted per pathophysiological domain. Each domain is represented along the x-and y-axes, labeled with the respective percentage of total variance explained by PC1 and PC2. The ellipse illustrates the central 10% of each MS1 group. Arrows in the plot indicate both the direction (arrow orientation) and magnitude (arrow length) of the correlation existing between each biomarker and the PCs. Adjacent to each PCA plot, boxplots facilitate group comparisons concerning PC1 and PC2. It is important to note that even a negative trend within a boxplot of a PC may denote a positive correlation with biomarker concentrations, as reflected by the direction of the arrows. Post-hoc analysis was conducted using a Tukey Test. Significance levels are represented as follows: ** p<0.0001, Abbreviations: IL: interleukin; MMP8: matrix metalloproteinase 8; NGAL: neutrophil gelatinase-associated lipocalin; ANG1: angiopoietin 1; ANG2: angiopoietin 2; sE-selectin: soluble E-selectin; sICAM-1: soluble intercellular adhesion molecule 1; PT: prothrombin time.


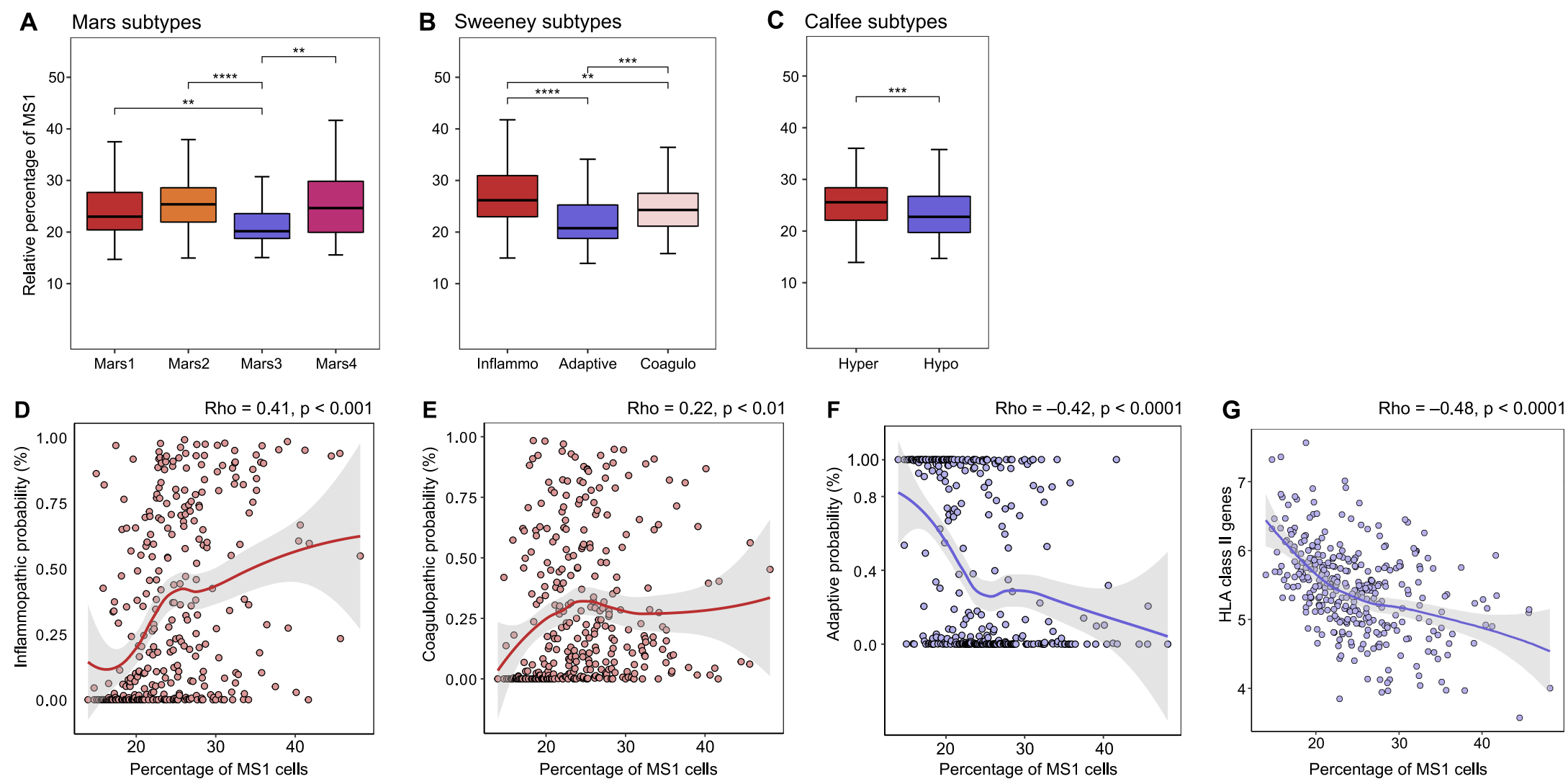


**Figure S8. Relation between MS1 cell proportions and previously described molecular subtypes and signatures in patients with sepsis**

**Description Figure S8: A)** Percentage of MS1 cells in Mars subtypes, **B)** Sweeney subtypes, and **C**) Calfee subtypes. Correlation analysis between the percentage of MS1 cells and **D)** inflammopathic, **E)** coagulopathic, **F**) adaptive probabilities, **G)** human leucocyte antigen (HLA) class II gene mean expression. Statistical analyses were performed using Mann-Whitney U or Kruskal-Wallis test with Dunn’s Post-Hoc Test corrected by Benjamini-Hochberg method. ****Dunn’s post-hoc test p<0.0001, *** p<0.001, ** p<0.01. Abbreviations: rho: Spearman correlation coefficient.


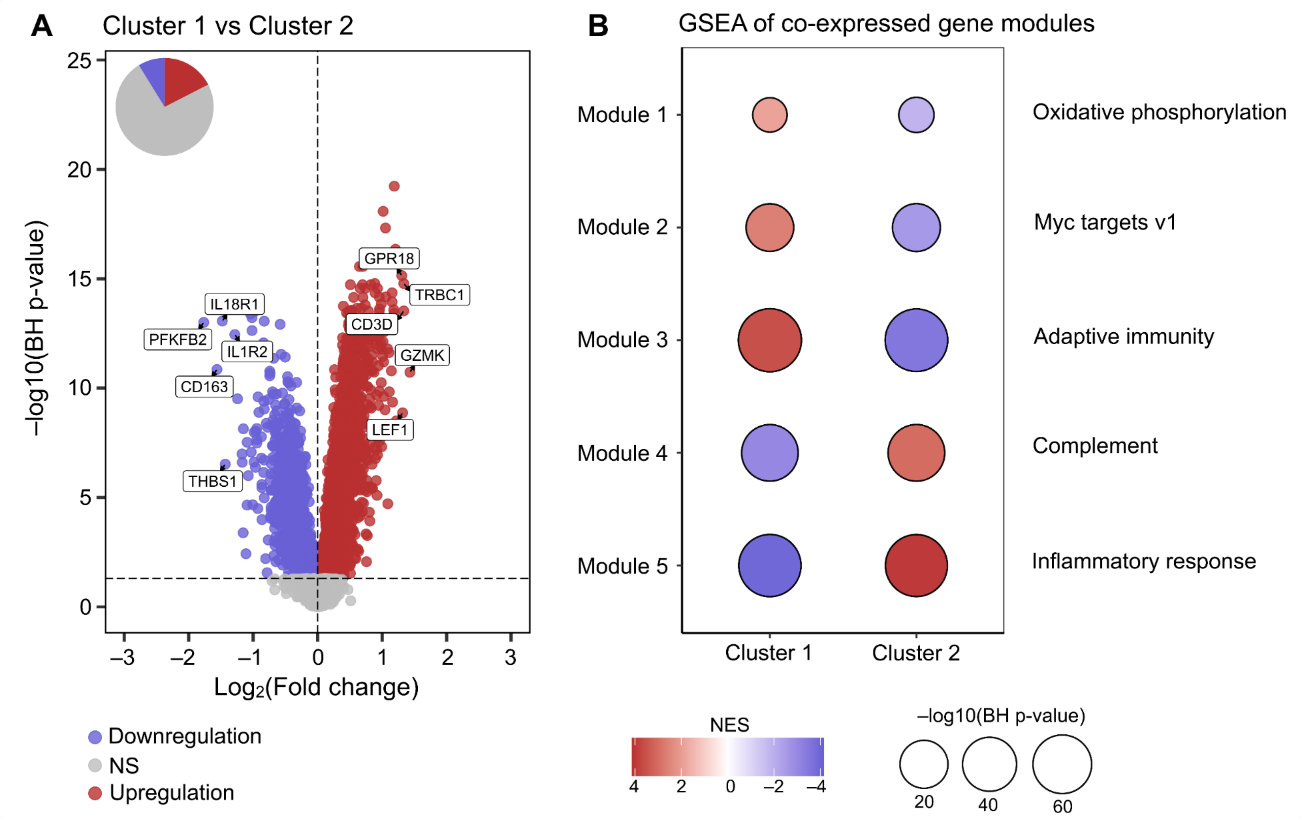


**Figure S9**. **Gene expression differences of non-infected critically ill patients stratified into clusters by percentage of MS1 cells using one-dimensional k-means clustering**

**Description Figure S9: A)** Volcano plots illustrating the differences in leukocyte genomic responses among patients classified as cluster 1 (low MS1) and cluster 2 (high MS1). Genes are identified as differentially expressed with a BH adjusted p-value of less than 0.05. Gene names indicate the top five upregulated (in red) and top five downregulated (in blue) genes. Gray dots represent genes that were not differentially expressed between groups. The pie chart shows the extent of gene expression differences. **B)** The co-expression module identification analysis revealed distinct gene modules based on MS1 cell levels in patients with sepsis. Patients were categorized into as cluster 1 (low MS1) and cluster 2 (high MS1). The size of each circle in the graph is proportional to -log10(BH adjusted p-value), and the color represents the normalized enrichment score (NES). Blue indicates a decreased NES, and red represents an increased NES.

**References**

1. Charlson ME, Pompei, P, Ales, KL, MacKenzie, CR. A new method of classifying prognostic comorbidity in longitudinal studies: development and validation. J Clin Epidemiol. 1987, 40(5),:373-383.

2. Singer M, Deutschman CS, Seymour CW, Shankar-Hari M, Annane D, Bauer M, et al. The Third International Consensus Definitions for Sepsis and Septic Shock (Sepsis-3). Jama. 2016, 315(8):801-810.

3. Bernard GR, Artigas A, Brigham KL, Carlet J, Falke K, Hudson L, et al. The American-European Consensus Conference on ARDS. Definitions, mechanisms, relevant outcomes, and clinical trial coordination. Am J Respir Crit Care Med. 1994, 149(3):818-824.

4. KDIGO AKI Work Group. KDIGO Clinical Practice Guideline for Acute Kidney Injury. Kidney Int Suppl 2012, 2:1-141.

5. Bellomo R, Ronco C, Kellum JA, Mehta RL, Palevsky P, et al. Acute renal failure - definition, outcome measures, animal models, fluid therapy and information technology needs: the Second International Consensus Conference of the Acute Dialysis Quality Initiative (ADQI) Group. Critical care. 2004, 8(4):R204-212.

6. Bourgon R, Gentleman R, Huber W. Independent filtering increases detection power for high-throughput experiments. Proc Natl Acad Sci U S A. 2010, 107(21):9546-9551.

7. Leek JT, Storey JD. Capturing Heterogeneity in Gene Expression Studies by "Surrogate Variable Analysis". PLOS Genetics. 2007, 3(9): e161.

8. Russo PST, Ferreira GR, Cardozo LE, Bürger MC, Arias-Carrasco R, Maruyama SR, et al: CEMiTool: a Bioconductor package for performing comprehensive modular co-expression analyses. BMC Bioinform. 2018, 19(1):56.

9. Liberzon A, Birger C, Thorvaldsdóttir H, Ghandi M, Mesirov JP, Tamayo P. The Molecular Signatures Database (MSigDB) hallmark gene set collection. Cell Syst. 2015, 1(6):417-425.

10. Cano-Gamez E, Burnham KL, Goh C, Allcock A, Malick ZH, Overend L, et al. An immune dysfunction score for stratification of patients with acute infection based on whole-blood gene expression. Sci Transl Med. 2022, 14(669):eabq4433.

11. Gonçalves ANA, Lever M, Russo PST, Gomes-Correia B, Urbanski AH, Pollara G, et al. Assessing the Impact of Sample Heterogeneity on Transcriptome Analysis of Human Diseases Using MDP Webtool. Front Genet. 2019, 10.

12. Sweeney TE, Azad TD, Donato M, Haynes WA, Perumal TM, Henao R, Bermejo-Martin JF, et al. Unsupervised Analysis of Transcriptomics in Bacterial Sepsis Across Multiple Datasets Reveals Three Robust Clusters. Crit Care Med. 2018, 46(6):915-925.

13. Davenport EE, Burnham KL, Radhakrishnan J, Humburg P, Hutton P, Mills TC, Rautanen A, et al. Genomic landscape of the individual host response and outcomes in sepsis: a prospective cohort study. Lancet Respir Med. 2016, 4(4):259-271.

14. Scicluna BP, van Vught LA, Zwinderman AH, Wiewel MA, Davenport EE, Burnham KL, Nurnberg P, et al: Classification of patients with sepsis according to blood genomic endotype: a prospective cohort study. Lancet Respir Med. 2017, 5(10):816-826.

15. Sinha P, Delucchi KL, McAuley DF, O'Kane CM, Matthay MA, Calfee CS. Development and validation of parsimonious algorithms to classify acute respiratory distress syndrome phenotypes: a secondary analysis of randomised controlled trials. Lancet Respir Med. 2020, 8(3):247-257.

16. Sinha P, Kerchberger VE, Willmore A, Chambers J, Zhuo H, Abbott J, Jones C, et al. Identifying molecular phenotypes in sepsis: an analysis of two prospective observational cohorts and secondary analysis of two randomised controlled trials. Lancet Respir Med. 2023.
